# Small RNA Library Preparation Method for Next-Generation Sequencing Using Chemical Modifications to Prevent Adapter Dimer Formation

**DOI:** 10.1371/journal.pone.0167009

**Published:** 2016-11-22

**Authors:** Sabrina Shore, Jordana M. Henderson, Alexandre Lebedev, Michelle P. Salcedo, Gerald Zon, Anton P. McCaffrey, Natasha Paul, Richard I. Hogrefe

**Affiliations:** 1 Research and Development, Cell and Molecular Biology, TriLink BioTechnologies LLC., San Diego, California, United States of America; 2 Engineering and Instrumentation, Synthetic Genomics, Inc., La Jolla, California, United States of America; USDA Agricultural Research Service, UNITED STATES

## Abstract

For most sample types, the automation of RNA and DNA sample preparation workflows enables high throughput next-generation sequencing (NGS) library preparation. Greater adoption of small RNA (sRNA) sequencing has been hindered by high sample input requirements and inherent ligation side products formed during library preparation. These side products, known as adapter dimer, are very similar in size to the tagged library. Most sRNA library preparation strategies thus employ a gel purification step to isolate tagged library from adapter dimer contaminants. At very low sample inputs, adapter dimer side products dominate the reaction and limit the sensitivity of this technique. Here we address the need for improved specificity of sRNA library preparation workflows with a novel library preparation approach that uses modified adapters to suppress adapter dimer formation. This workflow allows for lower sample inputs and elimination of the gel purification step, which in turn allows for an automatable sRNA library preparation protocol.

## Introduction

NGS is a powerful tool that yields vast amounts of sequence data and allows the analysis of many samples in parallel. Correlation with genetic databases allows identification of novel biomarkers for better diagnosis and more personalized treatment. Next-generation sequencing can be used to examine DNA or RNA for whole genome or transcriptome analysis, respectively. In addition, various techniques such as Chromatin Immunoprecipitation (ChIP), Cross-linking Immunoprecipitation (ClIP), and RNA Immunoprecipitation Sequencing (RIP-Seq) isolate protein/nucleic acid complexes and sequencing of the partitioned nucleic acids allows cataloging of molecular interactions. Transcriptome analysis can be further divided into long coding RNA (messenger RNA or mRNA), long non-coding RNA (lncRNA), or small RNA (sRNA). sRNA are < 500 nucleotides (nt) and consist of a variety of sRNA species including piwi interacting RNA (piRNA), small interfering RNA (siRNA), Y RNA, transfer RNA (tRNA), and mircroRNA (miRNA) [1–3]. miRNA are a well-studied class of 19–23 nt sRNAs which play a major role in gene regulation. With the discovery of circulating nucleic acids in blood samples, miRNA expression analysis in plasma or exosomes are becoming increasingly important for biomarker discovery in the diagnostics field [4]. While blood samples are less invasive to the patient than tissue biopsies, they contain very low levels of circulating RNA and are thus difficult to analyze. Therefore a highly sensitive method for sRNA analysis is crucial.

**Fig 1:**
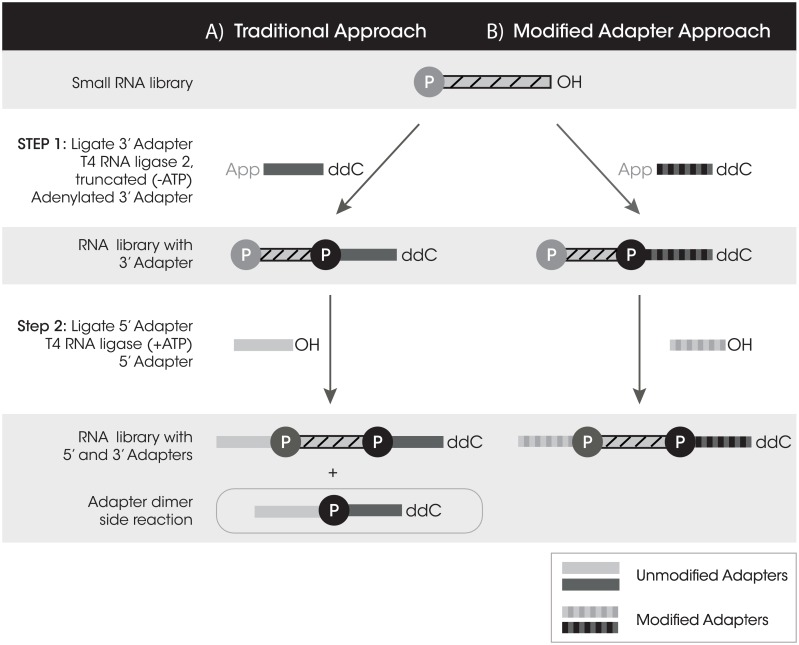
A comparison of small RNA library preparation workflows. A) The traditional approach with unmodified adapters which results in tagged library and adapter dimer. B) The modified adapter approach which results in primarily tagged library.

Long RNA-Seq and DNA-Seq library preparation techniques have matured quickly and allow PCR free library preparation with low sample inputs and automated protocols. Inherent obstacles for small RNA-Seq (sRNA-Seq) library preparation have thus far limited sequencing of lower RNA inputs and have prevented sRNA-Seq automation. In a traditional sRNA library preparation, oligonucleotides called adapters are ligated onto both the 5΄ and 3΄ ends of the small RNA targets (library) to form a tagged library pool (Fig 1A). These adapters provide a universal sequence used for downstream amplification of tagged libraries. The first ligation step requires a pre-adenylated 3΄ adapter and an RNA ligase lacking the ATP binding domain, which is specific for ligation between the adenylate to the 3΄ hydroxyl of an RNA or library insert. This feature of the RNA ligase prevents RNA library inserts from circularizing and self-ligating during this step. Additionally, the 3΄ adapter is blocked on its 3΄ end to prevent self-concatemerization. The second ligation step uses an RNA 5΄ adapter and an ATP dependent RNA ligase. The 5΄ adapter is not phosphorylated on the 5΄ end which prevents self-concatemerization, but during this second ligation step an unwanted side reaction of adapter dimer formation can occur when the 5΄ adapter ligates directly to any excess 3΄ adapter that has not already ligated to an RNA insert (Fig 1A). Since sRNA insert sizes are very short (~22 nt), the tagged library product is very similar in size to the undesired adapter dimer side product, a difference of approximately 20 nucleotides. This size similarity makes these two PCR products difficult to separate during purification so, a gel extraction step is required to isolate the tagged library away from adapter dimer. Furthermore, because the adapter dimer is smaller than the tagged library, its preferential amplification tends to dominate the downstream PCR reaction [5]. This problem is exacerbated at lower RNA inputs, where there is a very limited amount of library to tag but still substantial adapter dimer present. Using current commercially available kits at low inputs, tagged library becomes minimized (S1A Fig) while adapter dimer consumes the majority of the sequencing reads (S1B Fig).

Several methods have been developed to curtail adapter dimer formation. One method requires early inclusion of the reverse transcription (RT) primer, which is complementary to the 3΄ adapter. Directly after the first ligation step, the RT primer is added to create a double stranded product which cannot be ligated by T4 RNA Ligase 1 to the 5΄ adapter [6]. Other methods employ multiple purification steps to size select away excess adapters from the reaction to isolate desired products during the workflow [7]. An alternative strategy uses a non-ligation approach that entails 3΄ A-tailing of the library and a 5΄ template switching mechanism to prepare library ends for downstream RT and PCR. Elimination of the ligation steps precludes the formation of adapter dimer [3].

Gel purification is a common and often necessary size selection procedure as a final step in library preparation. However, even with a laborious gel purification step that can substantially reduce the amount of adapter dimer, its presence is not completely eliminated. Minimal amounts of adapter dimer contamination will be loaded onto the flow cell along with the tagged library and again preferentially amplify to form clusters and take up valuable sequencing reads that could otherwise be occupied by tagged library. Reproducibility of gel extractions can vary tremendously in percent recovery of sample and success largely depends on the technician performing the procedure. Furthermore, with low product recovery it is possible to lose RNA sequences that have low expression levels, leading to false negative data. Gel purification significantly limits the ability to automate library preparation for sRNA thus limiting high throughput experiments. The two most common commercially available kits, TruSeq Small RNA Library Preparation Kit (Illumina) and NEBNext Small RNA Library Prep Set (New England Biolabs), recommend 100 ng total RNA input as the lowest sample amount achievable [8]. Below 100 ng of total RNA input, it becomes challenging to produce high quality sequencing data, which hinders sequencing of low input samples such as plasma or urine.

We have developed a novel small RNA library preparation method which uses chemically modified adapters to prevent adapter dimer formation by blocking ligation of the 5΄ and 3΄ adapters to one another, while allowing for efficient tagging of adapters onto the small RNA library (Fig 1B). Chemical modifications can be introduced onto oligonucleotides to enhance enzymatic reactions or to prevent specific reactions from occurring until desired. These chemical modifications can be placed on the sugar, the base, or the inter-nucleotide phosphate linkages. In the present study, we specifically investigate the ligation step of the library preparation workflow since that is the source of adapter dimer formation. We screened 256 different combinations of modifications to determine how they influenced ligase function. Our results show that certain combinations of modifications completely inhibit ligation activity, while some enhance ligation efficiency, and yet others have a variable effect. The combination of one modification near the ligation junction on the 3΄ adapter and a different modification on the 5΄ adapter proved to suppress adapter dimer formation while allowing for adapter tagging of the sRNA library to occur. We also reasoned that when two chemical modifications were in close proximity (as they would be in the adapter dimer) they would prevent reverse transcriptase (RT) read-through during the cDNA synthesis step. In contrast when the two chemical modifications were separated by a library insert (tagged library), they would permit RT read-through. Thus adapter dimer would be limited at both the ligation and cDNA synthesis steps. With the suppression of adapter dimer, a gel purification step is no longer required and can be replaced with a two-step automatable bead based size selection, allowing for the sample to be directly loaded onto a sequencer. sRNA library preparation using modified adapters extends the current limits of detection (100 ng) to ultra-low inputs or single cell quantities (10 pg) of total RNA [5]. Here we demonstrate that rendering adapter dimer formation negligible is the key to overcoming a major challenge for sRNA library preparation.

## Materials and Methods

### Oligonucleotides

Modified adapter oligonucleotides and PCR primers were synthesized at TriLink BioTechnologies, LLC. All oligonucleotides were purified by polyacrylamide gel electrophoresis (PAGE). P = 5΄ phosphate; ddC = 2΄,3΄-dideoxycytidine; rApp = adenylate; MP = methylphosphonate; 2΄OMe = 2΄-O-methyl.

LuoDon R FAM: 5΄ FAM-GTACCAGTCGCCTAGAATACT-3΄

Luo 3΄ Adapter (51nt): 5΄ **(P)AGTTGTCATAGTTTGATCCTCTAGTCTGG**GAGTATTCTAGGCGACTGGTAddC 3΄

Original sequence taken from Luo et al. paper in bold. Primer binding site added to end (underlined) [9]

Unmodified 3΄ Adapter: 5΄ (rApp)TGGAATTCTCGGGTGCCAAGG(ddC) 3΄

Unmodified 5΄ Adapter: 5΄ GUUCAGAGUUCUACAGUCCGACGAUC 3΄

CleanTag 3΄ Adapter: 5΄ (rApp)T(MP)GGAATTCTCGGGTGCCAAGG(ddC) 3΄

CleanTag 5΄ Adapter: 5΄ GUUCAGAGUUCUACAGUCCGACGAU (C) 2΄OMe 3΄

Alternate 3΄ Adapter Sequence: 5´rAppA(MP)GATCGGAAGAGCACACGTCT-NH2–3´

RT primer: 5΄ GCCTTGGCACCCGAGAATTCCA 3΄

Forward primer: 5΄ AATGATACGGCGACCACCGAGATCTACACGTTCAGAGTTCTACAGTCCGA 3΄

Reverse primer: (barcode sequence underlined, interchangeable with Index 1–24) 5΄ CAAGCAGAAGACGGCATACGAGATCGTGATGTGACTGGAGTTCCTTGGCACCCGAGAATTCCA 3΄

Illumina compatible barcoded reverse PCR primers:

Index Primer set 1 for Illumina (Catalog #L-3204 TriLink BioTechnologies, LLC): Index 1: CGTGAT, Index 2: ACATCG; Index 3: GCCTAA; Index 4: TGGTCA; Index 5: CACTGT; Index 6: ATTGGC; Index 7: GATCTG; Index 8: TCAAGT; Index 9: CTGATC; Index 10: AAGCTA; Index 11: GTAGCC; Index 12: TACAAG.

Index Primer set 2 for Illumina (Catalog #L-3205 TriLink BioTechnologies, LLC): Index 13:TTGACT; Index 14:GGAACT; Index 15:TGACAT; Index 16:GGACGG; Index 17:CTCTAC; Index 18:GCGGAC; Index 19:TTTCAC; Index 20:GGCCAC; Index 21:CGAAAC; Index 22:CGTACG; Index 23:CCACTC; Index 24:GCTACC.

Let7d-3p (NNN): 5' P-NNNUACGACCUGCUGCCUUNNN 3'

Let7d-5p (NNN): 5' P-NNNGGUAGUAGGUUGCAUANNN 3΄

Let-7d-3p RNA: 5' P-CUAUACGACCUGCUGCCUUUCU 3'

Let-7d-5p RNA: 5' P-AGAGGUAGUAGGUUGCAUAGUU 3΄

### Ligation reactions

Initial ligation reactions were performed with higher concentrations of adapters (each ~ 1μM) and synthetic RNA (1μM) for visualization purposes. Ligation conditions for each ligase are as listed in library preparation section below unless otherwise indicated. 1X tris/borate/ethylenediaminetetraacetic acid (TBE)-Urea buffer (ThermoFisher) was added in equal volumes to ligation reactions. Samples were heated for 2 minutes at 70°C prior to loading onto a 10% TBE-Urea Novex precast gel (ThermoFisher). Gel was run at 165 volts for approximately 25 minutes and then stained for 5 minutes with SYBR Gold (ThermoFisher). Gel densitometry was performed using ImageJ to quantify product bands [10].

### cDNA synthesis through modifications

Ligation: 1X T4 RNA Ligase buffer, 1 mM ATP, 10% DMSO, 20% PEG, 20 Units RNase Inhibitor, 20 Units of T4 RNA Ligase 1, 20 μM 3’ Adapter Luo, 20 μM 5’ Adapter, 10 μM Let-7d-3p RNA, 20 μL total volume. Incubated at 22°C for 2 hours. After ligation, EDTA was added to 10 mM final concentration followed by 20 μL of TBE-Urea. 30 μL of each sample was heated for 3 minutes at 72°C prior to loading onto a 12% PAGE 7M Urea gel. Gel was run at 1200 volts for approximately 2 hours or until the bromephenol blue ran to the bottom. Bands were cut out of the gel and left in 0.3 M NH4OAc over night at room temperature. Samples were then ethanol precipitated and prepared for cDNA synthesis. Sample concentrations were estimated based on the gel image and approximate equal volumes of each RNA was used per respective reaction. 1X First Strand cDNA synthesis buffer, 40 Units RNase Inhibitor, 1μM of LuoDon R FAM, 0.5 mM dNTPs, 200 Units SuperScript II reverse transcriptase, 15 μL total volume. Samples were incubated at 50°C for 50 minutes then 85°C for 5 minutes and imaged. Gel densitometry was performed using ImageJ to quantify product bands and all results were normalized to unmodified adapter dimer.

### Library preparation

#### CleanTag Small RNA Library Preparation Kit (Catalog #L-3206 TriLink BioTechnologies, LLC.)

Ligation Step 1: 1X Buffer 1 (50 mM Tris(hydroxymethyl)aminomethane HCl pH 7.5, 10 mM MgCl_2_, 1 mM dithiothreital, ~20% polyethylene glycol (PEG) 8000) (TriLink Biotechnologies), 0.5 μM CleanTag 3΄ Adapter (TriLink Biotechnologies, LLC.), 40 Units murine RNase Inhibitor (New England Biolabs), 200 Units of T4 RNA Ligase 2 truncated KQ (New England Biolabs), RNA input (1 μg), 10 μL total volume. Denature RNA at 70°C for 2 minutes before use. Incubate total reaction 1 hour at 28°C then heat inactivate enzyme for 20 minutes at 65°C. Ligation Step 2: 3΄ tagged library from step 1, 1X Buffer 2 (50 mM Tris·HCl pH 7.5, 10 mM MgCl_2_, 1 mM DTT, 2 mM ATP) (TriLink BioTechnologies, LLC.), 2 μM CleanTag 5΄ Adapter (TriLink BioTechnologies, LLC.), 40 Units RNase Inhibitor (New England Biolabs), 20 Units of T4 RNA Ligase 1 (New England Biolabs), 20 μL total volume. Heat 5΄ adapter for 2 minutes at 70°C then combine with reaction. Incubate 1 hour at 28°C then heat inactivate for 20 minutes at 65°C. Reverse Transcriptase Step: 20 μL of tagged library from step 2, 1X Protoscript II reverse transcription buffer (New England Biolabs), 8 mM DTT (New England Biolabs), 40 Units RNase Inhibitor (New England Biolabs), 200 Units Protoscript II Reverse Transcriptase (New England Biolabs), 0.3 μM Reverse Transcription Primer (TriLink BioTechnologies, LLC.), 0.4 mM deoxyribonucleotide triphosphates (dNTPs) (TriLink BioTechnologies,LLC.), 36 μL total volume. Combine RT primer with tagged library and heat at 70°C for 2 minutes, then add remaining reagents to reaction. Incubate 1 hour at 50°C. PCR Step: RT product, 1x High Fidelity PCR Master Mix (New England Biolabs), 0.5 μM Forward primer (TriLink BioTechnologies, LLC.), and 0.5 μM Reverse Index Primer (TriLink BioTechnologies, LLC.), 80 μL total volume. Denature at 98°C for 30 seconds then cycle 12X [98°C for 10 seconds, 60°C for 30 seconds, 72°C for 15 seconds] followed by final extension at 72°C for 10 minutes. For RNA inputs below 1 μg the 3΄ and 5΄ adapter concentrations and PCR cycles were adjusted depending on RNA input amount: 1000 ng total RNA (1X adapters; 12 PCR cycles), 100 ng (1:2 dilution of adapters; 15 PCR cycles), 10 ng (1:4; 18), 1 ng (1:12; 21), 100 pg (1:14; 24), 10 pg (1:16; 27). These parameters were optimized for samples with high quality RNA.

#### Alternative enzymes used during preliminary optimizations and experiments

Ligases: T4 RNA Ligase 2, truncated (New England Biolabs) and T4 RNA Ligase 2, truncated K227Q (New England Biolabs). Other RT enzymes: Superscript II Reverse Transcriptase (ThermoFisher).

#### RNA inputs

A number of commercially available RNA templates were used for these studies. FirstChoice Human Brain total RNA and Breast Adenocarcinoma (MCF-7) Total RNA are available from Life Technologies (Catalog # AM7962 and # AM7846, respectively). Universal Human Reference RNA (UHR) is composed of total RNA from 10 human cell lines including adenocarcinoma (mammary gland), hepatoblastoma (liver), adenocarcinoma (cervix), embryonal carcinoma (testis), glioblastoma (brain), melanoma, liposarcoma, histiocytic lymphoma (macrophage and histocyte), lymphoblastic leukemia (T lymphoblast), and plasmacytoma/myeloma (B lymphocyte) (Agilent, catalog #740000). Human trachea total RNA is available from Agilent Technologies (catalog #540145). T Helper Cell (CD4+) total RNA can be purchased from Miltenyi Biotec (catalog #130-093-163). miRXplore Universal Reference (Miltenyi Biotec #130-093-521) is a commercially available pool of 963 synthetic miRNA (human, mouse, rat, virus) at equimolar quantities of 5 fmol/μL.

### Analysis

#### Gel electrophoresis

Libraries (crude or purified) were visualized by 4% EX agarose E-gel (Invitrogen Catalog G4010-04) run for 30 minutes and imaged with UV transilluminator.

#### Agilent 2100 Bioanalyzer analysis

Library prep samples after PCR (crude or purified) were analyzed by 2100 (Agilent Technologies). Samples were diluted 1:10 with water and then 1 μL was loaded onto a High Sensitivity DNA chip for analysis. The 2100 Expert software was used to quantitate library peaks. For smear analysis all peaks within regions 100–300 nt were included in quantification.

### Purification

Size selection targeting 100–200 nucleotides was performed on small RNA libraries using Agencourt AMPure XP beads (Beckman Coulter).1X AMPure XP magnetic beads (80 μL) was added to 80 μL of final PCR product then mixed and incubated at room temperature for 10 minutes. Tubes were then placed on magnetic rack for 4 minutes to separate the beads. Supernatant containing desired product was then transferred to a new clean tube and 1.8X original PCR volume (144 μL) of beads were mixed and incubated with supernatant for 10 minutes at room temperature. Again, tubes were placed on magnetic rack for 4 minutes to separate beads and supernatant. This time, supernatant was discarded and beads were washed twice with 500 μL of 70% ethanol on magnetic rack. Beads were then air dried for 5 minutes on magnetic rack before resuspended in 17 μL nuclease free water. Beads were incubated in water for 2 minutes at room temperature before placed back on magnetic rack for elution. 15 μL of eluate was collected as purified product.

Gel purification was performed on PCR samples run on 4% EX agarose E-Gels (Invitrogen Catalog G4010-04) using Zymoclean Gel DNA Recovery Kit (Zymo Research Catalog D4007).

### Library multiplexing and pooling

In most cases, experiments were planned to contain 24 samples which would be barcoded, pooled equally, and then loaded onto one lane of a flow cell. Libraries were prepared individually and barcoded with reverse primers during the PCR step which contained Illumina compatible indices #1–24. Individual library concentrations were determined by running a high sensitivity Bioanalyzer chip followed by smear analysis. Since all samples were to occupy a similar amount of space on the flow cell, we divided the final concentration of our pooled mixture, 10 nM for example, by 24 to determine that each samples final concentration should equal 0.416 nM. We then calculated the volume of each sample that would be required to meet this number based on the individual concentration. Each of the 24 samples was pooled accordingly to occupy equal space on a single lane of a flow cell.

### Sequencing

All next-generation sequencing experiments were performed on an Illumina HiSeq 2500 with single-end 100 nt reads. 24 samples were loaded onto one lane of a flow cell. All sequencing was outsourced to a core facility (TSRI- Scripps Research Institute) or company (GENEWIZ).

### Data analysis

Original FASTQ sequencing files are deposited in the NCBI Short Reads Archive (SRA) under Bioproject number PRJNA350292, SRA study number SRP092414 (SRA accession numbers: SRX2317287-7296; SRX23177298-7314; SRX23177316-7318; SRX23177321-7330; SRX23177330-7346). Data analysis was performed using a number of different methods and in some cases outsourced, as described below. See figure and table legends for analysis method used. Initial NGS runs were outsourced to a core facility at TSRI, with data analysis and statistics performed by their in-house bioinformatician. Subsequent data sets were analyzed using Geneious version R8 from Biomatters [11, 12] or the UC Davis public Galaxy AMI toolshed. Data analysis with the UC Davis Galaxy software [13] used the following tools and workflow: FastQC (v0.11.2) [14], Clip (v 1.1.1) where the following adapter sequence was identified and clipped: TGGAATTCTCGGGTGCCAAGG and sequences shorter than 15 nt were discarded. Sickle (7667f147e6) [15] was performed using single end reads with a quality threshold of 30 and length threshold of 15. MiRDeep2 Mapper (version 2.0.0.5) [16, 17] was used to collapse reads. MiRDeep2 Quantifier was used to map reads to miRBase mature human miRNA sequences (release 21) [18–25] with one allowed mismatch. Reads were also mapped to piRBase version 1.0 Database 2014; doi: 10.1093/database/bau110 [26]. Comparisons were made between Geneious software and Galaxy tools to make sure similar results were obtained throughout experiments. In some cases, BaseSpace (Illumina) [27] was used to generate figures. Statistical analyses were performed in Graphpad with one-way Anova (Tukeys multiple comparison test).

## Results

### Chemically modified adapters suppress adapter dimer

Adapter chemical modifications screened consisted of either sugar modifications at the n, n-1, or n-2 nucleotide sugars or backbone modifications at the n, n-1, or n-2 inter-nucleotide phosphate linkages. Sugar modifications included 2'-fluoro (F), 2'-O-methyl (OMe), and 2'-deoxy-2'-fluoro-beta-D-arabinonucleic acid (FANA). Backbone modifications included phosphorothioate (Ps) and methylphosphonate (MP) (S2 Fig). While there were only 5 modifications, there were three different positions near the ligation junction so each modification would yield 2–3 adapters to test. In some cases, two modifications were used on the same adapter. We designed matrices that combined one of the modified 3' adapters with each of the modified 5' adapters and vice versa producing 256 combinations of modifications (S1 Table).These combinations of modifications were interrogated with the following criteria in mind: 1) ability to ligate to an unmodified RNA library; 2) inhibition of 5΄ and 3΄ adapter ligation to prevent adapter dimer; and 3) ability of reverse transcriptase enzymes to read through modifications, when separated by an RNA insert, to form an unmodified cDNA for downstream PCR.

The effect of modifications on ligation was initially determined by assessing the yield of ligation reactions using one modified adapter, one unmodified adapter, and T4 RNA Ligase 1. These yields were compared to ligations which used an unmodified version of both the adapters. All modified 3΄ adapters produced a ligation product when ligated to an unmodified RNA oligonucleotide (5΄ Adapter) (S3A Fig). Most modifications did not reduce ligation yields when compared to unmodified with the exception of the MP (n-1) modification (S3A Fig). There was more variability in yields with modifications on the 5΄ adapter (S3B Fig). Most modified 5΄ adapters ligated efficiently to another unmodified oligonucleotide (3΄ Adapter Luo) with the exception of MP modifications and one of the OMe modifications, all of which had reduced yields (S3B Fig). An MP at the first inter-nucleotide linkage on the 5´ adapter was the only modification that failed to produce a detectable product at the ligation step. Later, ligation efficiency experiments were repeated with a different 3΄ adapter (Unmodified 3΄ adapter) and a synthetic Let7d RNA oligonucleotide for the library insert. Ligation yields varied slightly with new sequences, however overall patterns remained consistent (data not shown).

**Fig 2:**
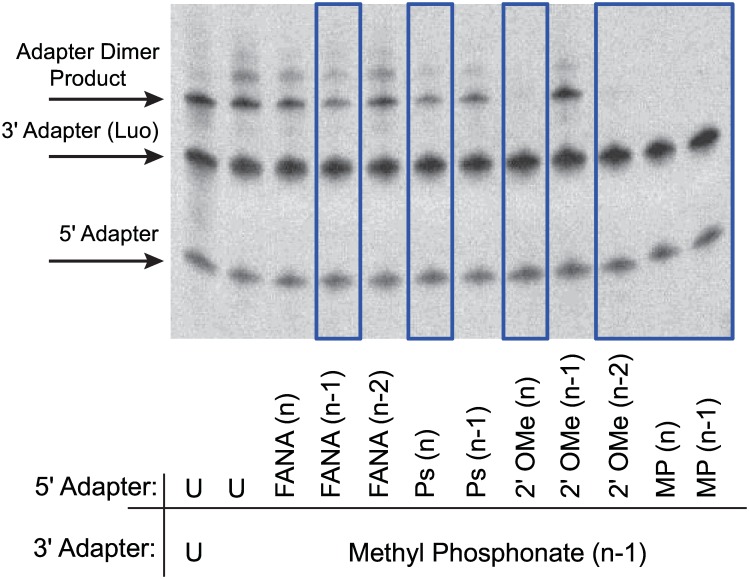
Ligation screen for modified adapters that suppress adapter dimer formation. Example of modifications screened on the 5´adapter for ligation suppression against the Luo 3΄ Adapter with MP (n-1). Unmodified adapters were shown for comparison (U = unmodified). Adapter concentrations were 1 μM. Ligations performed with 10 U T4 RNA Ligase 1, 1 mM ATP, and 20% PEG, incubated for 2 hours at 37°C. Candidate modifications which reduce dimer formation are highlighted with blue box.

Next, ligation reactions were performed with T4 RNA ligase 1 in the absence of target library RNA with combinations of modified 5΄ adapters and modified 3΄ adapters to discern which pairs had reduced ligation yields, a measure of prevention of adapter dimer formation. It was determined early on that there was only one modified 3΄ adapter (MP at the n-1 position) that worked to prevent ligation when paired with other modified 5΄ adapters (data not shown). All other modifications when placed on the 3΄ adapter exhibited very little effect to suppress ligation. We therefore focused on modified 3΄ adapter MP (n-1) moving forward. Several combinations with various modifications on the 5΄ adapter reduced adapter dimer formation (FANA (n-1), PS (n)) and a few seemed to completely inhibit it (2΄Ome (n), 2΄Ome (n-2), MP (n), MP (n-1)) (Fig 2). From this, a smaller group of promising modified adapter combinations was tested in sequential ligation steps to assess yield of tagged library and adapter dimer. In general, results showed that modified adapters which suppressed adapter dimer produced lower yields for tagged libraries than their unmodified versions (data not shown) and therefore key components in the ligation workflow were further optimized.

**Fig 3:**
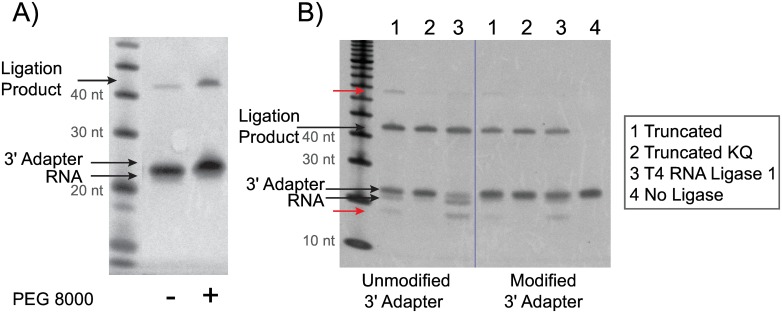
Optimization of the 3´ adapter ligation step. Synthetic Let-7d-5p (NNN) miRNA was ligated to the 3´ adapter using the same ligation conditions as the CleanTag library prep workflow step 1. A) Yield increase with addition of PEG 8000 using T4 RNA Ligase 2, truncated KQ and modified 3´ adapter (MP (n-1)). B) Specificity comparison between ligases used in 3´ ligation step: 1) T4 RNA Ligase 2, truncated; 2) T4 RNA Ligase 2, truncated KQ; 3) T4 RNA Ligase 1; 4) No Ligase. Both unmodified and modified (MP (n-1)) 3´ adapters were tested. Side products indicated with red arrows.

Several aspects of the library preparation workflow were evaluated to determine if they would increase library ligation yield while maintaining specificity. Optimizations included adapter concentration, incubation temperatures and times, ligation buffers, ATP concentration, polyethylene glycol (PEG) percentage, and ligase enzymes. There were several critical components which improved the overall ligation yield significantly. We determined that the most critical ingredient for increased ligation yields was PEG 8000. Optimal PEG concentration of 18.75% allowed for a significant increase in yield for the 3´ adapter ligation step (Fig 3A). A 4-fold excess of 5΄ adapter concentration over the 3΄ adapter also increased yields (data not shown). Initially we compared several T4 RNA Ligase enzymes in the first ligation step: T4 RNA Ligase 1 and several truncated T4 RNA Ligase 2 derivatives. T4 RNA Ligase 1 is an ATP dependent single stranded RNA ligase which can ligate single stranded RNA or DNA oligonucleotides [3]. While T4 RNA Ligase 1 is typically only used in the second ligation step, we found that this enzyme could also be used in the first ligation step with an adenylated oligonucleotide in the absence of ATP. The truncation derivatives of T4 RNA Ligase 2 allow for ATP independent ligation on single stranded RNA or RNA/DNA hybrids. The truncation derivatives tested include T4 RNA Ligase 2, truncated (T42t); T4 RNA Ligase 2, truncated K227Q; and T4 RNA Ligase 2, truncated KQ [28]. Preliminary results indicated that K227Q offered no advantage for ligation yield or specificity (data not shown) so this enzyme was excluded early on. In a fully optimized workflow the ligation yields between three of the enzymes were comparable, however the use of KQ resulted in a more specific ligation product, so this ligase was used in subsequent experiments (Fig 3B). After all ligation components were optimized, the modified adapters still produced slightly lower yields at the ligation step than the unmodified adapters, however, due to the reduction in adapter dimer formation, downstream library yields after PCR were increased.

**Fig 4:**
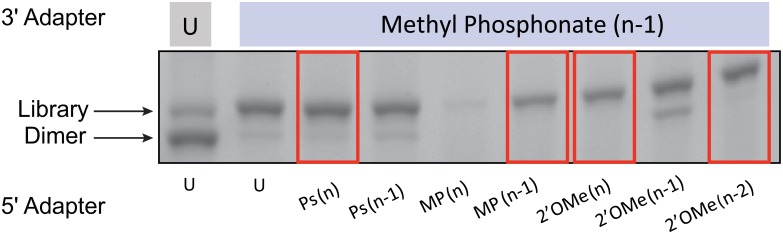
Screen for the best combination of modified adapter pairs for suppression of adapter dimer. Top combinations of modified adapters were tested in a full CleanTag library prep workflow from ligation to RT-PCR for dimer suppression. 0.7 ng Let-7d-3p (NNN) synthetic miRNA input. Samples run on a 4% agarose gel stained with ethidium bromide. Best combinations are shown in red boxes. U = unmodified.

**Fig 5:**
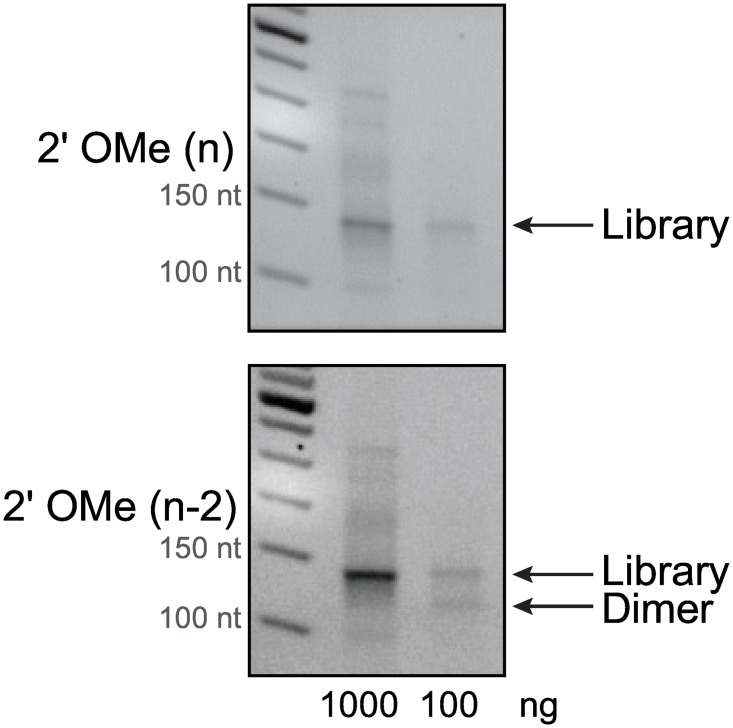
Investigation of modified adapter combinations at lower RNA inputs. Brain total RNA at 1000 or 100 ng input was tested with candidate modified adapters in a full library preparation workflow. The modified 3´ adapter was MP (n-1) and the modified 5´ adapter was either 2´ OMe (n) or 2´ OMe (n-2). Agarose gel analysis of the product from 12 cycles of PCR. No adapter dilutions were made.

Next, a full library prep workflow including the reverse transcription (RT) and PCR step was performed with various promising modified adapter combinations (Fig 4). Results indicated that reverse transcription was possible with most modifications when an insert RNA was present between them (Fig 4) but specific yields of solely the RT step for all modifications were not assessed. Modified adapter combinations which provided the highest library yield with the lowest amount of adapter dimer (MP (n-1) on the 3´ adapter paired with PS (n), MP (n-1), 2´OMe (n), or 2´OMe (n-2) on the 5´ adpater) were further investigated for performance at lower levels of RNA input. Not surprisingly, the MP (n) modification on the 5´ adapter resulted in low library yield as we had previously observed undetectable ligation product when tested with an unmodified oligonucleotide (S3B Fig). Preliminary studies with an RNA input of 1000 ng of total brain RNA revealed almost complete suppression of adapter dimer for both the OMe (n) and the OMe (n-2) modifications (Fig 5). However when using ten-fold lower total RNA input (100ng), adapter dimer was no longer suppressed by the OMe (n-2) modification while the OMe (n) modification continued to show suppression (Fig 5).

**Fig 6:**
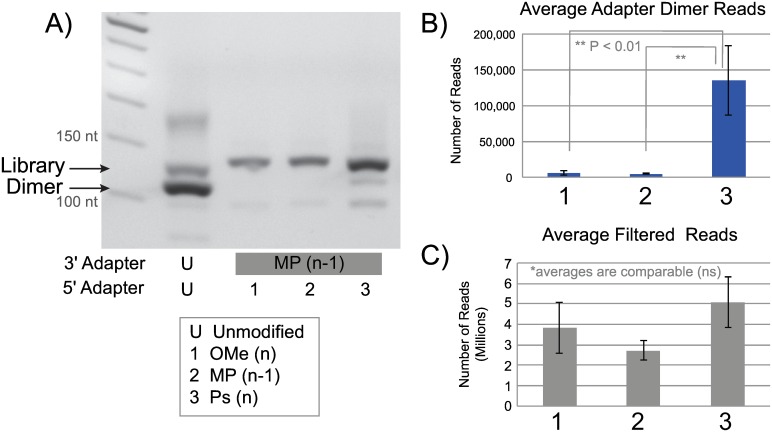
Next-generation sequencing run comparing top modified adapter combinations. Libraries prepared with unmodified or modified (MP(n-1)) 3´ adapter and unmodified or various modified 5´ adapter (1 = OMe(n), 2 = MP (n-1), or 3 = Ps(n)) using a pool of 963 synthetic miRNA (Miltenyi) following CleanTag library preparation protocol. Data analysis performed by TSRI. A) Agarose gel analysis of crude library PCR products. Sequencing Data: B) Average number of adapter dimer reads, C) Average number of filtered reads.

Next, three top combinations of modified adapters were used in a full library preparation workflow on a synthetic miRNA pool (Miltenyi) and then sequenced in an NGS run to dissect out any minimal differences between the modifications (Fig 6). The PS modification (combination 3) displayed increased library yield by gel, while producing just a slight amount of adapter dimer (Fig 6A). However the data from the NGS run had significantly more adapter dimer reads than the other modified adapters but a statistically comparable amount of filtered (mappable) reads (Fig 6B and 6C). Ultimately this modification did not offer any downstream advantage by having more library yield to begin with. This further proves that even small levels of adapter dimer present in the reaction can be exacerbated when clustering on the flow cell. The other two combinations of adapters preformed comparably but further experimentation revealed more consistent results for combination 1: MP at the n-1 position on the 3΄ adapter and an OMe modification on the n position of the 5΄ adapter (Fig 6). This combination of modified adapters is now referred to as CleanTag. The final CleanTag adapters with modifications are the Illumina compatible CleanTag 3΄ adapter [5΄-(rApp)T(MP)GG AAT TCT CGG GTG CCA AGG (ddC)- 3΄] and the Illumina compatible CleanTag 5΄ adapter [5΄- GUU CAG AGU UCU ACA GUC CGA CGA UC(OMe)-3΄].

Finally, we investigated whether these modifications, when in close proximity without an RNA insert (adapter dimer), would inhibit the cDNA synthesis step. This in turn would further help to suppress any residual adapter dimer if formed at the ligation step. The ability to reverse transcribe through a single MP and a single OMe modification was evident since a product was formed from a tagged library but there was no specific investigation into whether these modifications slowed down cDNA synthesis. To test the effect of the modifications on reverse transcription for the CleanTag modifications, we used a FAM labeled RT primer to track cDNA synthesis yield on various adapter dimer versions: A) unmodified; B) 3΄ adapter modified only; C) 5΄ adapter modified only; and D) both 3΄ and 5΄ modified adapters. At very high concentrations and with nothing else in the reaction to compete with, we were able to force ligation of the modified adapters to one another and conduct the downstream RT reaction. Reading through two modifications in close proximity to each other proved challenging for the reverse transcriptase enzyme as cDNA synthesis was decreased by 70% (S4 Fig). This gave a clear indication that any residual ligated adapter dimer would further be suppressed during the RT step when using modified adapters.

We then introduced our top modifications onto another set of adapter sequences but interestingly, we determined that adapter dimer suppression was not as dramatic as when using the CleanTag adapter sequences (S5 Fig). No further investigation was done to determine why the modifications only worked within specific adapter sequences.

### Effect of modifications on the tagged library population

**Fig 7:**
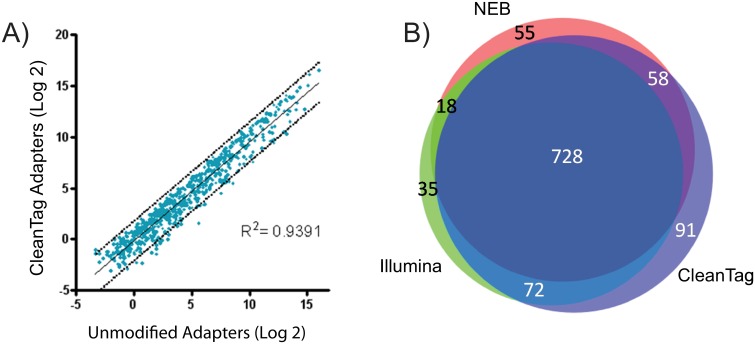
Effect of adapter modifications on tagged library population. Libraries were prepped with 1000 ng human brain total RNA and the CleanTag library prep protocol or recommended manufacturers conditions for Illumina and NEB kits. Data analysis performed by TSRI. A) Correlation plot of unmodified adapters and modified CleanTag adapters within the CleanTag library prep. Tagged miRNA are plotted after Log2 transformation. B) Venn diagram of CleanTag kit, Illumina TruSeq kit, and NEBNext kit depicting number of brain miRNA identified in all 3 replicates for each workflow.

In order to determine if the chemical modifications introduced any significant changes in miRNA detection, we compared sequencing results between unmodified and modified adapters within our optimized workflow. We found that a similar population of miRNA was tagged by unmodified adapters compared to those tagged by the modified adapters (Fig 7A). Though there are slight differences between the two libraries, this provided evidence that within our workflow the modifications themselves were not significantly skewing the tagged miRNA population. Furthermore, a comparison across multiple commercial kits showed that each kit tags specific miRNA that the other kits do not, however, the majority (728 miRNA) of the tagged miRNA population were similar amongst all three kits (Fig 7B).

### Lower inputs

**Fig 8:**
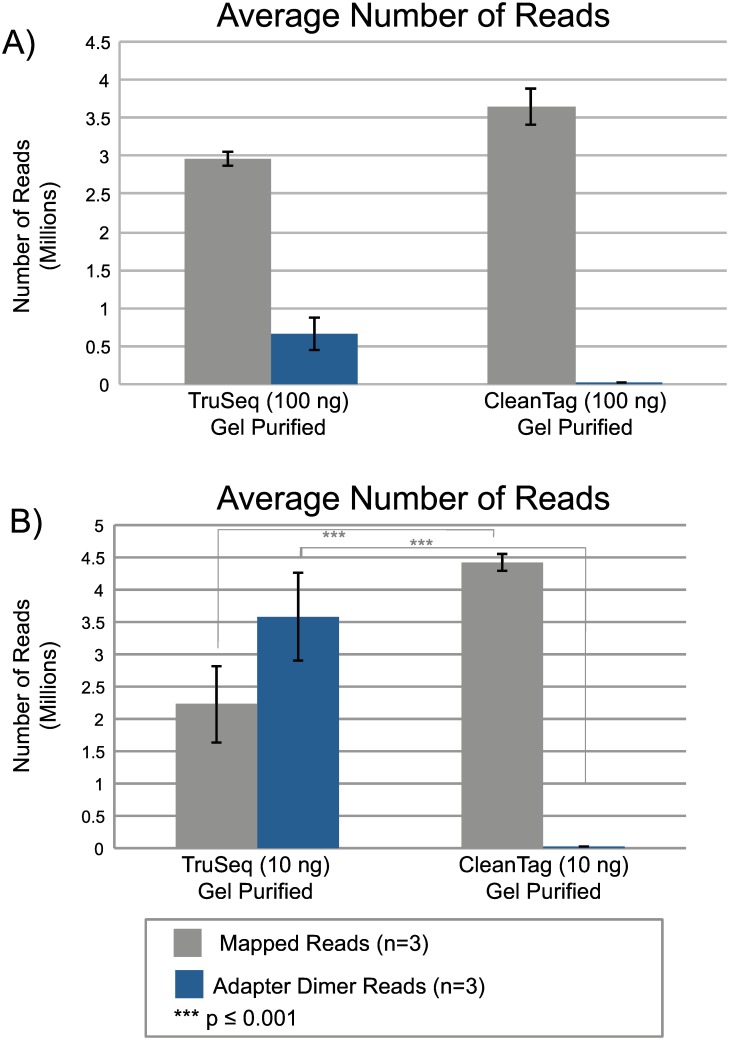
NGS data comparison between CleanTag and TruSeq Small RNA Library Preparation Kit. Libraries prepared with TruSeq Small RNA Library Preparation Kit or CleanTag workflow with total human brain RNA input and gel purification. Samples sequenced on a HiSeq 2500 SR, 1x 100bp. Human total brain RNA at A) 100 ng, or B) 10 ng input. Data analysis performed using Geneious. Statistical analysis performed with GraphPad-One way ANOVA Turkeys multiple comparison test.

**Fig 9:**
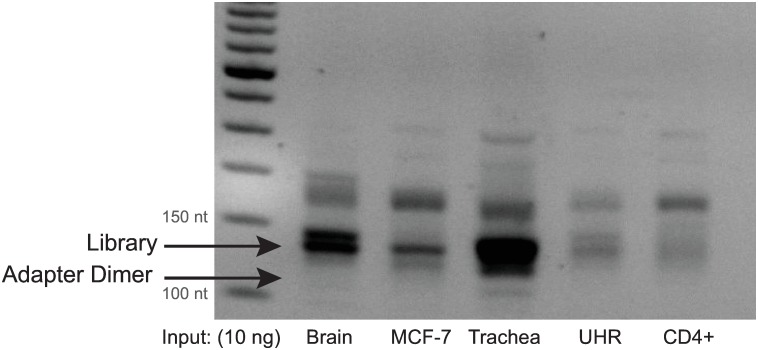
Agarose gel analysis of PCR purified libraries with various total RNA inputs at 10ng. Libraries prepared with CleanTag workflow. UHR is Universal Human Reference RNA.

With strong suppression of adapter dimer using CleanTag modifications in the library preparation workflow, sequencing from much lower RNA inputs is now possible. Samples using a range of human total brain RNA inputs were sequenced to determine the limit of detection using the modified adapters. Adapters were diluted to optimized concentrations for each amount of total RNA input and PCR cycles were increased accordingly (Materials and Methods, Sample Preparation Section). Samples prepared with modified adapters were compared to the TruSeq small RNA Library Preparation Kit (Illumina). The TruSeq kit recommends a minimum of 1000 ng RNA input in combination with a gel purification step after library preparation; however, we also tested this kit at lower RNA inputs. When analyzing these two workflows at 100 ng total RNA input using a gel purification step, mapped reads and adapter dimer reads were statistically comparable (Fig 8A). However, at 10 ng total RNA input, the samples prepared with CleanTag adapters yielded significantly more mapped reads and significantly less adapter dimer than the TruSeq kit (Fig 8B). Even with a tedious gel purification step, the TruSeq samples lost 48% of their reads to adapter dimer at 10 ng compared to less than 1% when using modified adapters. With 1 nanogram total RNA input using CleanTag adapters, we found less than 1% of reads lost to adapter dimer while sustaining similar levels of mappable reads compared to the higher inputs (S2 Table). This demonstrates that lower RNA inputs can now achieve quality sequencing results without losing copious reads to adapter dimer. Furthermore, we have tested a number of total RNAs extracted from various cell lines that vary in the amount of miRNA they contain to ensure the protocol is robust for many sample types and samples with low abundance of miRNA (Fig 9).

### Gel-free clean up for automated library prep

**Fig 10:**
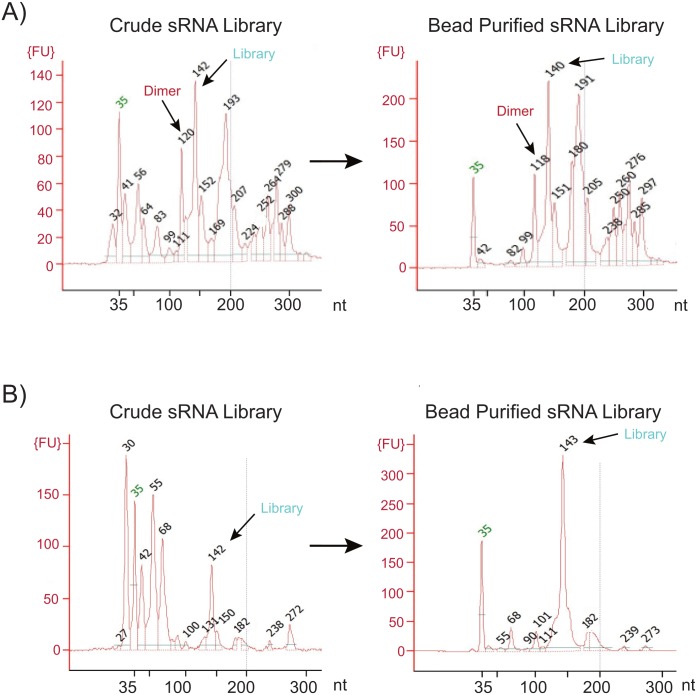
Comparison of crude and bead purified libraries using CleanTag or TruSeq small RNA library prep kit. Bioanalyzer traces of libraries prepared using 1000 ng human total brain RNA input. Crude or AMPure XP purified PCR products with A) TruSeq small RNA library preparation kit, or B) CleanTag small RNA library preparation kit.

**Fig 11:**
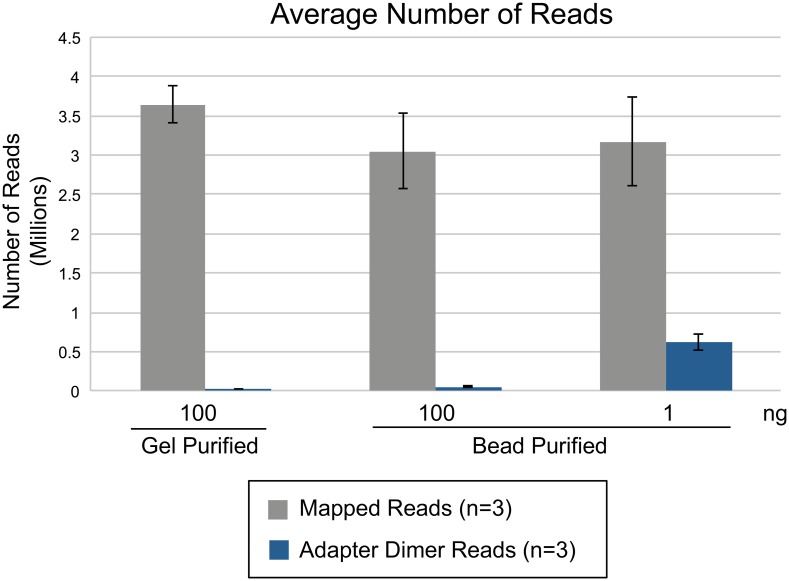
Comparison of NGS data between gel purified and bead purified samples within a CleanTag workflow. Libraries prepared with CleanTag small RNA library prep kit and human brain total RNA input. PCR samples were purified by gel extraction or 2-step AMPure XP bead-based protocol. Data analysis was performed with Geneious.

In addition to allowing for lower RNA inputs, removal of the adapter dimer also eliminates the need for the gel purification step. This in turn facilitates automation of the small RNA library prep process. A two-step AMPure XP bead-based purification protocol was optimized to size select product between 100–200 nucleotides. Library preparation with the CleanTag workflow results in limited adapter dimer but also a decrease in side products. This leads to a cleaner bead purified sample downstream, especially when compared to TruSeq Small RNA Library Prep Kit (Fig 10). While bead-based purification does not completely isolate the library of interest, CleanTag produces a trace where the miRNA library is the major peak or product while everything else remains at background level. Despite the fact that other products are also loaded onto the flow cell, NGS data revealed no loss in total number of miRNA reads from bead purified libraries when compared to a gel purified sample at 100-fold higher input (Fig 11). The number of miRNA identified was also comparable between purification methods (S2 Table). In addition, we did a thorough investigation into individual peaks which appear on a Bioanalyzer trace after bead-based purification to determine their origin (S1 File). The ability for automation with bead-based protocols enables higher throughput sequencing for faster data acquisition in both research and diagnostic settings.

### Single cell inputs

**Fig 12:**
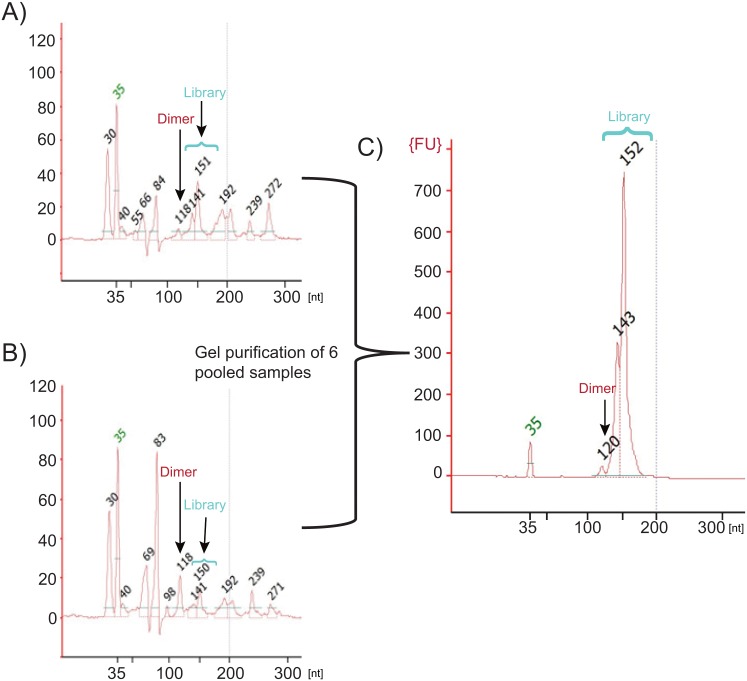
Single cell quantities of small RNA can be tagged for next-generation sequencing. Example (one of three replicates) bioanalyzer traces of crude PCR product libraries prepped using the CleanTag library prep workflow with human brain total RNA at A) 100 pg for 24 PCR cycles, or B) 10 pg inputs for 27 PCR cycles. C) Gel purified pool of ultra-low input (3 replicates of 100 pg and 3 replicates of 10 pg) samples.

**Fig 13:**
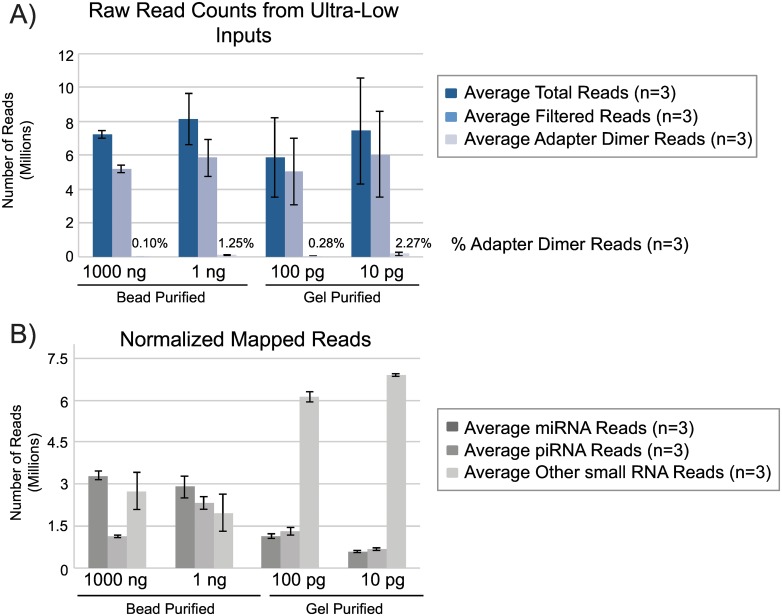
Next-generation sequencing data with single cell quantities of small RNA. NGS data analysis of samples prepared with human brain total RNA inputs at 1000ng, 1ng, 100pg, and 10pg. 1000ng and 1ng samples were bead-purified and 100pg and 10pg samples were pooled and gel purified. Data analysis was performed using Galaxy. A) Raw read counts of total reads, filtered reads (after 3´adapter trimming and quality filtering), and adapter dimer reads. B) Normalized mapped read counts for small RNA types: miRNA, piRNA, other small RNA.

**Fig 14:**
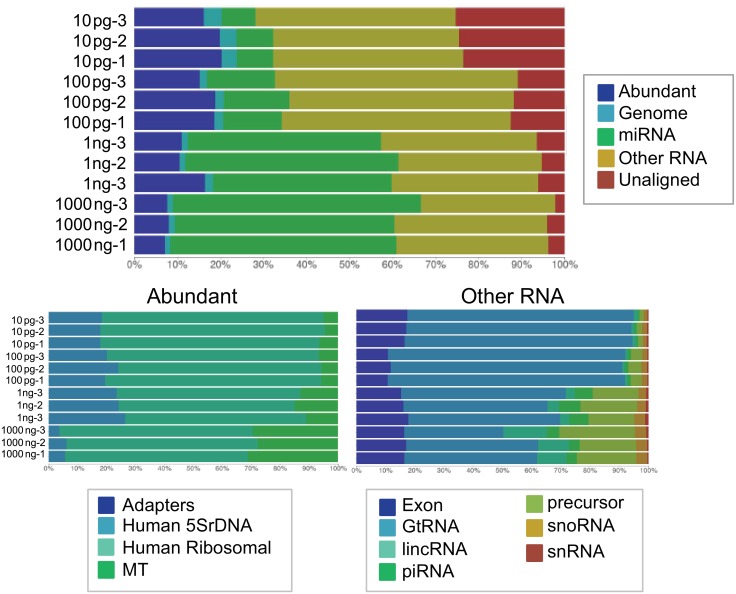
Distribution of tagged small RNA in ultra low input libraries. Small RNA libraries were prepared with modified adapters using various amounts of human brain total RNA input, sequenced on a HiSeq2500, and analyzed using BaseSpace sRNA App. The abundant categories and small RNA categories were further subcategorized.

We further pushed the limit of detection for small RNA library prep using modified adapters down to 100 and 10 pg of human brain total RNA (Fig 12A and 12B). A single cell has approximately 10 pg total RNA. Adapter dilutions and PCR cycles were adjusted accordingly for each input amount. While a small amount of adapter dimer is now evident at these ultra low input levels there is still an adequate amount of library to sequence when previously these amounts of RNA inputs gave no detectable library. In order to extract the best quality sequencing results from these ultra low inputs, all replicates of the 100 and 10 pg samples (three samples each) were pooled and gel purified to reduce any low levels of adapter dimer product (Fig 12C). Higher input samples (1000 and 1 ng) were prepared using a bead-based purification for comparison to ultra low inputs at 100 and 10 pg which were prepared using gel purification. Each input group consisted of three replicates and libraries were pooled to generate similar amounts of reads across samples. The total number of reads and downstream filtered reads (3΄ adapter trimmed and quality filtered) were comparable across all sample inputs (Fig 13A). The reads lost to adapter dimer were less than 3% even at 10 pg input. Filtered reads were mapped to miRbase mature miRNA database and piRNA database using Galaxy to generate individual read counts per sample. All filtered reads that were not mapped to miRbase or piRNA database were considered “other small RNA” (Fig 13B). The percentage of small RNA types (miRNA, piRNA, other) were later confirmed by analysis with BaseSpace (Illumina) which further categorized the other small RNAs (Fig 14). At 1000 and 1 ng of RNA input, 46% and 40% of the reads respectively were attributed to miRNA, a trend we observed initially where mapping quality was fairly consistent amongst inputs. At the 100 and 10 pg levels, mapped miRNA reads fall to 13% and 7%, respectively (S3 Table). A closer analysis of the tagged miRNAs in each sample revealed that lower input samples maintain reads of highly expressed miRNA in brain (S4 Table). Read counts for top 40 expressed miRNA contain previously validated miRNA enriched in brain including miR-9, miR-128, and Let 7 family members, [29–32] while lower abundance miRNA tend to drop out at lower inputs of 100 and 10 pg. Another trend that was observed with ultra low inputs is that 50% of the reads were now dominated by “other” small RNA of which the majority consisted of tRNA. It is unclear why these species begin to dominate the workflow as the RNA input drops and further investigation is ongoing.

## Discussion

### Chemically modified adapters suppress adapter dimer

Herein we have demonstrated the use of oligonucleotide modifications to specifically inhibit the ligation of the 5΄ adapter to the 3΄ adapter thereby significantly reducing adapter dimer formation and improving the specificity of small RNA library preparation workflows. The specific interaction of a 2΄ OMethyl (OMe) and a methyl phosphonate (MP) modification on the adapters proved to be one of few combinations which inhibited ligation and furthermore repressed RT read through and downstream amplification. While certain other modifications initially appeared to suppress ligation, after RT-PCR, the adapter dimer ligation product could be detected and was an indication that not all modifications that suppressed ligation had the added benefit of preventing RT read through. We therefore chose to optimize the workflow around the top combination of modified adapters that suppressed adapter dimer to the greatest extent after both the ligation and reverse transcription-PCR steps. Although initial ligation yields using modified adapters were slightly lower than unmodified adapters, upon workflow optimization the benefit of reduced adapter dimer seemed to help promote RT and PCR amplification of the tagged library and diminish any lower yield effects brought on by the modifications during ligation.

Interestingly, the adapter dimer suppression effect seen with our top modifications seems to depend largely on the sequence of the adapters. These modifications were later tested on several other sets of adapter sequences (data from one example shown) but the same level of adapter dimer suppression was not achieved (S5 Fig). Simply changing the primary sequence of either of the adapters results in higher yields of adapter dimer formation which indicates there may be an underlying sequence effect. While the reason for this remains unclear, we speculate secondary structure and folding within the ligase active domain may be part of the cause. The modifications on the adapters are not the sole reason for significant reduction of dimer. The workflow for using the modified adapters was optimized to improve yield and specificity of small RNA library formation. Initially library yields were lower in comparison to unmodified adapters. Extensive testing of individual components, reagents, and incubation times were investigated thoroughly. PEG concentration was determined to be one of the critical components that when optimized had a significant effect on library yield. Recovery of ligation yield through increased PEG levels has been previously observed [3, 28]. PEG is thought to act as a molecular crowding reagent that increases the local concentrations of adapter and library. Furthermore, the modified adapters cannot be easily substituted into other workflows or commercially available kits. It is therefore a combination of the modified adapters and optimized conditions which act to prevent adapter dimer formation.

### Modifications do not alter tagged library population

It is known that RNA ligases have specific preferences for their substrates, thus introducing a level of bias into the small RNA library preparation workflow [3]. It is clear that many current library preparation protocols may not cover all small RNA species due to inherent secondary structures of miRNA [33, 34]. We investigated whether the chemically modified adapters would significantly alter miRNA signatures when compared to unmodified adapters in the same workflow. Given that the modifications did not tag a different population of miRNA compared to unmodified adapters further investigation was not done.

### Lower inputs

A limitation of current small RNA library preparation is high input requirements. Low RNA inputs often result in high adapter dimer yield as this product is preferentially formed and amplified. This makes investigation of small RNAs from precious biological samples difficult as they often give low RNA yield. We have demonstrated that small RNA sequencing from as low as 1 ng total RNA input is made possible due to suppressed adapter dimer. We were unable to produce sufficient library at such low inputs using commercially available kits, as adapter dimer was the dominant product, which emphasizes the importance of dimer suppression, even when gel purification is used. At 1 ng total RNA input we obtained quality sequencing data without significant loss of miRNA reads. At these input levels more limiting biological samples can be easily interrogated. Lower RNA inputs will expand the small RNA-Seq field in several ways. Small RNA from biofluids (plasma, serum, urine, saliva), FACS cells, exosomes, Clip-Seq, and FFPE samples can more easily be analyzed by next-generation sequencing with higher quality sequencing data. Examples of sRNA library preparation with CleanTag and these challenging sample types are presented elsewhere [35].

We found that moving to lower RNA input levels also required the dilution of the adapter input in order to maintain a reduction in adapter dimer formation. Very low amounts of adapter were needed to form tagged libraries at low RNA inputs. With the reduction in RNA input, an increase in number of PCR cycles was needed to generate enough copies of tagged library for downstream sequencing.

### Gel-free clean up for automated library preparation

Automated high throughput sample preparation that is common for DNA-Seq or RNA-Seq has not yet been implemented for small RNA sequencing. This is largely due to the post library preparation gel clean up needed to eliminate adapter dimer. With the suppression of adapter dimer formation an automatable bead-based purification method can replace manual gel extractions. In general our workflow using modified adapters results in cleaner small RNA libraries with less side products as compared to other commercial kits. Therefore bead purification of CleanTag libraries is a sufficient clean up method for sequencing at most routine RNA inputs. While bead purified samples contain a variety of tagged species which can also be sequenced, we showed that this did not significantly detract from the number of mappable miRNA reads in the sample. Sequencing data generated from bead purified samples produced comparable number of miRNA reads to that of gel purified samples despite the presence of additional tagged species. With the elimination of adapter dimer there is more functional space on the flow cell for sequencing other important small RNA targets in addition to miRNA. miRNA are often still the dominant and shortest product in the reaction so these will preferentially amplify over any other larger molecular weight species, thus maintaining number of mappable reads for this important small RNA category.

While maintaining miRNA information, bead-based methods also offers the ability to analyze all types of sRNA tagged in a library ranging in size from 100 to 200 nt, not just a specific type or length of sRNA such as miRNA that has been size selected from a gel. Different cell types or bodily fluids may contain different RNA signatures and be enriched in other types of sRNA other than miRNA. Valuable information including new small RNA biomarkers can be extracted from these sample types and a more in depth analysis can be done when data from other small RNA would have been previously excluded by gel excising the 140 nt targets exclusively. As further information is gained and various new types of sRNA are discovered, small RNA-Seq is becoming increasingly important. Now, more information can be gained from sequencing bead-purified samples and analyzing all small RNA species that are present in a given sample.

It is now possible for liquid handling robots to be programmed for automated small RNA library preparation and purification to prepare samples for direct loading onto the sequencer. This will significantly decrease hands on time, human error, and increase throughput. CleanTag improves small RNA sequencing by 1) enabling sequencing of samples containing as little as 1 ng total RNA; 2) allowing for automation by the use of a bead-based purification; and 3) increasing throughput potential with an automated workflow.

### Single cell quantity inputs

Samples with ultra low levels of RNA including single cell quantities (10 pg) of material can now be easily analyzed by NGS. Preliminary results reveal adequate amounts of library generated from these low inputs, a sufficient amount of quality reads generated after filtering, but an overall loss in low expressed miRNA species and an overabundance of tRNA reads. The biological relevance of this observation remains to be examined. The importance of tRNA fragments as other small RNA biomarkers is increasing, especially in breast cancer research [2, 36, 37]. It has yet to be demonstrated whether this approach could currently be useful to specifically interrogate biomarkers which are overexpressed in certain diseases or cancers at these ultra low input levels. Further investigation remains to be done to improve the process and increase number of miRNA reads at single cell levels.

## Conclusions

We have demonstrated that rendering adapter dimer formation negligible overcomes many of the current challenges for sRNA library preparation. There are multiple benefits from adapter dimer suppression including the feasibility of using ultra-low total RNA inputs, potential to automate the entire workflow, and elimination of a gel-extraction clean up step. These improvements significantly enhance sRNA library preparation workflow and the ability to sequence ultra low inputs now opens up sRNA-Seq to more sample types regardless of limiting material. This includes single cell samples, FACS sorted cells, FFPE, Clip-Seq, biological fluids, etc. This modified adapter technology may also be applied to other library preparation techniques such as long RNA-Seq, and cell free ssDNA-Seq in the near future, as our investigations of these applications is ongoing.

## Supporting Information

S1 Fig Examples of current input limitations with small RNA library preparation. A) 4% agarose gel analysis of bead purified PCR products. Libraries were prepared with unmodified adapters and 0.7 to 70 ng of a synthetic miRNA (Let 7d-3p (NNN)). B) NGS data showing average number of mapped reads and average number of adapter dimer reads. Libraries were prepared with the recommended conditions of the TruSeq Small RNA Library Prep Kit using unmodified adapters and brain total RNA at inputs of 10, 100, and 1000 ng. Data analysis was performed using Geneious.
(TIF)

S2 Fig Adapter chemical modifications. Representative chemical modifications screened on adapter oligonucleotides for sRNA-Seq library preparation. Nomenclature for the positions modified is shown.
(TIF)

S3 Fig Ligation efficiency for modifications on 5´ and 3´ adapters. Example of screened modifications on A) 3´ adapter Luo or B) the 5´ adapter for ligation efficiency. Red box indicates a modification in which ligation to the substrate was undetectable. Reactions were incubated with 10 U T4 RNA Ligase 1 for 1 hour at 37°C.
(TIF)

S4 Fig Effect of modification proximity on reverse transcription yield. Reverse transcription was performed on different modified adapter dimer substrates using a FAM-labeled RT primer. Ligation product from unmodified adapters served as the control for normalization (column A). Read through from a single modification on either the 3´ adapter (B) or 5´ adapter (C) was compared to a double modified substrate (D), both 5´ and 3´ adapters modified. RT products were run on a gel, imaged, and quantified for relative cDNA synthesis yield determination.
(TIF)

S5 Fig Library preparation comparison using top modifications on two different sets of adapter sequences. A) Library preparation using 7 ng synthetic miRNA (Let 7d-3p (NNN)) input. U = both adapters were unmodified; M = both adapter were modified with top modifications. The CleanTag adapter set was compared to an alternate adapter set with a different sequence for the 3´ adapter. The same modifications were used for the alternate set. B) The alternate adapters were also tested with 1000 ng brain total RNA input. The CleanTag library preparation workflow was used to prepare libraries.
(TIF)

S1 File Analysis of BioAnalyzer peaks generated from sRNA library preparation. (PDF)

S1 Table List of all 256 combinations of modifications screened for adapter dimer suppression. (XLSX)

S2 Table NGS table of sequencing results for low input. Comparison of data between different small RNA library preparations using human brain total RNA input between 1–100 ng. Libraries sequenced on HiSeq 2500 SR, 1x 100bp. Data analysis was performed using Geneious. Samples run in triplicate.
(TIF)

S3 Table NGS table summary of sequencing results for ultra-low input. Libraries prepared with human brain total RNA at various input amounts using CleanTag library preparation kit. All samples performed in triplicate. Inputs at 1000 and 1 ng were bead purified. Inputs at 100 and 10 pg were pooled and gel purified. Libraries sequenced on HiSeq 2500 SR, 1x 100bp and data analysis was performed with Galaxy.
(TIF)

S4 Table miRNA counts table for ultra-low input samples. Breakdown of data from S3 Table shows the average total number of miRNA detected at each input and the average raw read counts for the top 40 expressed miRNA at each input. Data analysis was performed with Galaxy.
(TIF)

## References

[1] Lopez JP, Diallo A, Cruceanu C, Fiori LM, Laboissiere S, Guillet I, et al Biomarker discovery: quantification of microRNAs and other small non-coding RNAs using next generation sequencing. BMC Med Genomics. 2015;8:35 10.1186/s12920-015-0109-x 26130076 PMC4487992

[2] Vickers KC, Roteta LA, Hucheson-Dilks H, Han L, Guo Y. Mining diverse small RNA species in the deep transcriptome. Trends Biochem Sci. 2015;40(1):4–7. 10.1016/j.tibs.2014.10.009 25435401 PMC4362530

[3] Raabe CA, Tang TH, Brosius J, Rozhdestvensky TS. Biases in small RNA deep sequencing data. Nucleic Acids Res. 2014;42(3):1414–26. 10.1093/nar/gkt1021 24198247 PMC3919602

[4] Turchinovich A, Weiz L, Langheinz A, Burwinkel B. Characterization of extracellular circulating microRNA. Nucleic Acids Res. 2011;39(16):7223–33. 10.1093/nar/gkr254 21609964 PMC3167594

[5] Head SR, Komori HK, LaMere SA, Whisenant T, Van Nieuwerburgh F, Salomon DR, et al Library construction for next-generation sequencing: overviews and challenges. Biotechniques. 2014;56(2):61–4, 6, 8, passim. 10.2144/000114133 24502796 PMC4351865

[6] Larry A. McReynolds DM, inventor; New England Biolabs, Inc., assignee. Method for Reducing Adapter-Dimer Formation patent US20150072870 A1. Mar 12, 2015.

[7] Technologies ITbL. Ion Total RNA-Seq Kit v2 User Guide2013.

[8] Inc. NEB. NEBNext Small RNA Library Prep Set for Illumina (Multiplex Compatible)2015.

[9] Luo J, Bergstrom DE, Barany F. Improving the fidelity of Thermus thermophilus DNA ligase. Nucleic Acids Res. 1996;24(15):3071–8. 8760896 10.1093/nar/24.15.3071 PMC146030

[10] Rasband WS. ImageJ 1997–2016 [Available from: http://imagej.nih.gov/ij/.

[11] Geneious—Beautiful bioinformatics tools for DNA, RNA, protein analysis 2016 [Internet]. Available from: http://www.geneious.com.

[12] Kearse M, Moir R, Wilson A, Stones-Havas S, Cheung M, Sturrock S, et al Geneious Basic: an integrated and extendable desktop software platform for the organization and analysis of sequence data. Bioinformatics. 2012;28(12):1647–9. 10.1093/bioinformatics/bts199 22543367 PMC3371832

[13] Afgan E, Baker D, van den Beek M, Blankenberg D, Bouvier D, Cech M, et al The Galaxy platform for accessible, reproducible and collaborative biomedical analyses: 2016 update. Nucleic Acids Res. 2016;44(W1):W3–W10. 10.1093/nar/gkw343 27137889 PMC4987906

[14] Andrews S. FastQC A Quality Control tool for High Throughput Sequence Data [Available from: http://www.bioinformatics.babraham.ac.uk/projects/fastqc/.

[15] Joshi NA F J. Sickle: A sliding-window, adaptive, quality-based trimming tool for FastQ files (Version 1.33) 2011 [Available from: https://github.com/majoshi/sickle.

[16] Friedlander MR, Mackowiak SD, Li N, Chen W, Rajewsky N. miRDeep2 accurately identifies known and hundreds of novel microRNA genes in seven animal clades. Nucleic Acids Res. 2012;40(1):37–52. 10.1093/nar/gkr688 21911355 PMC3245920

[17] Mackowiak SD. Identification of novel and known miRNAs in deep-sequencing data with miRDeep2. Curr Protoc Bioinformatics. 2011;Chapter 12:Unit 12 0. 10.1002/0471250953.bi1210s36 22161567

[18] miRBase sequence database version 9.2 [Available from: http://microrna.sanger.ac.uk/.

[19] Griffiths-Jones S. The microRNA Registry. Nucleic Acids Res. 2004;32(Database issue):D109–11. 10.1093/nar/gkh023 14681370 PMC308757

[20] Griffiths-Jones S. miRBase: the microRNA sequence database. Methods Mol Biol. 2006;342:129–38. 10.1385/1-59745-123-1:129 16957372

[21] Griffiths-Jones S. miRBase: microRNA sequences and annotation. Curr Protoc Bioinformatics. 2010;Chapter 12:Unit 12 9 1–0. 10.1002/0471250953.bi1209s29 20205188

[22] Griffiths-Jones S, Grocock RJ, van Dongen S, Bateman A, Enright AJ. miRBase: microRNA sequences, targets and gene nomenclature. Nucleic Acids Res. 2006;34(Database issue):D140–4. 10.1093/nar/gkj112 16381832 PMC1347474

[23] Griffiths-Jones S, Saini HK, van Dongen S, Enright AJ. miRBase: tools for microRNA genomics. Nucleic Acids Res. 2008;36(Database issue):D154–8. 10.1093/nar/gkm952 17991681 PMC2238936

[24] Kozomara A, Griffiths-Jones S. miRBase: integrating microRNA annotation and deep-sequencing data. Nucleic Acids Res. 2011;39(Database issue):D152–7. 10.1093/nar/gkq1027 21037258 PMC3013655

[25] Kozomara A, Griffiths-Jones S. miRBase: annotating high confidence microRNAs using deep sequencing data. Nucleic Acids Res. 2014;42(Database issue):D68–73. 10.1093/nar/gkt1181 24275495 PMC3965103

[26] Zhang P, Si X, Skogerbo G, Wang J, Cui D, Li Y, et al piRBase: a web resource assisting piRNA functional study. Database (Oxford). 2014;2014:bau110. 25425034 10.1093/database/bau110 PMC4243270

[27] Welcome—BaseSpace Sequence Hub 2016 [Internet]. Available from: https://basespace.illumina.com/.

[28] Song Y, Liu KJ, Wang TH. Elimination of ligation dependent artifacts in T4 RNA ligase to achieve high efficiency and low bias microRNA capture. PLoS One. 2014;9(4):e94619 10.1371/journal.pone.0094619 24722341 PMC3983213

[29] Biomarkers of Brain Injury and Neurological Disorders: CRC Press Taylor and Francis Group; 10 28, 2014.

[30] Hu HY, Guo S, Xi J, Yan Z, Fu N, Zhang X, et al MicroRNA expression and regulation in human, chimpanzee, and macaque brains. PLoS Genet. 2011;7(10):e1002327 10.1371/journal.pgen.1002327 22022286 PMC3192836

[31] Landgraf P, Rusu M, Sheridan R, Sewer A, Iovino N, Aravin A, et al A mammalian microRNA expression atlas based on small RNA library sequencing. Cell. 2007;129(7):1401–14. 10.1016/j.cell.2007.04.040 17604727 PMC2681231

[32] Shao NY, Hu HY, Yan Z, Xu Y, Hu H, Menzel C, et al Comprehensive survey of human brain microRNA by deep sequencing. BMC Genomics. 2010;11:409 10.1186/1471-2164-11-409 20591156 PMC2996937

[33] Fuchs RT, Sun Z, Zhuang F, Robb GB. Bias in ligation-based small RNA sequencing library construction is determined by adaptor and RNA structure. PLoS One. 2015;10(5):e0126049 10.1371/journal.pone.0126049 25942392 PMC4420488

[34] Romaniuk E, McLaughlin LW, Neilson T, Romaniuk PJ. The effect of acceptor oligoribonucleotide sequence on the T4 RNA ligase reaction. Eur J Biochem. 1982;125(3):639–43. 7117259 10.1111/j.1432-1033.1982.tb06730.x

[35] Shore Sabrina, Henderson Jordana M., Quintana Juan F., Chow Franklin W.N., Chhatbar Kashyap, McCaffrey Anton P., Zon Gerald, Buck Amy, Hogrefe Richard I. Improving NGS Small RNA Discovery in Biological Fluids and Other Low Input Samples. Keystone Symposia- Small RNA Silencing: Little Guides, Big Biology (A6); 1 24–28; Keystone, CO2016.

[36] Dhahbi JM, Spindler SR, Atamna H, Boffelli D, Martin DI. Deep Sequencing of Serum Small RNAs Identifies Patterns of 5' tRNA Half and YRNA Fragment Expression Associated with Breast Cancer. Biomark Cancer. 2014;6:37–47. 10.4137/BIC.S20764 25520563 PMC4260766

[37] Goodarzi H, Liu X, Nguyen HC, Zhang S, Fish L, Tavazoie SF. Endogenous tRNA-Derived Fragments Suppress Breast Cancer Progression via YBX1 Displacement. Cell. 2015;161(4):790–802. 10.1016/j.cell.2015.02.053 25957686 PMC4457382

