# Supplementary figures and images for: Small RNA Library Preparation Method for Next-Generation Sequencing Using Chemical Modifications to Prevent Adapter Dimer Formation
Source: PLoS One. 2016 Nov 22;11(11):e0167009. doi: 10.1371/journal.pone.0167009 (PMC5119831; doi:10.1371/journal.pone.0167009)

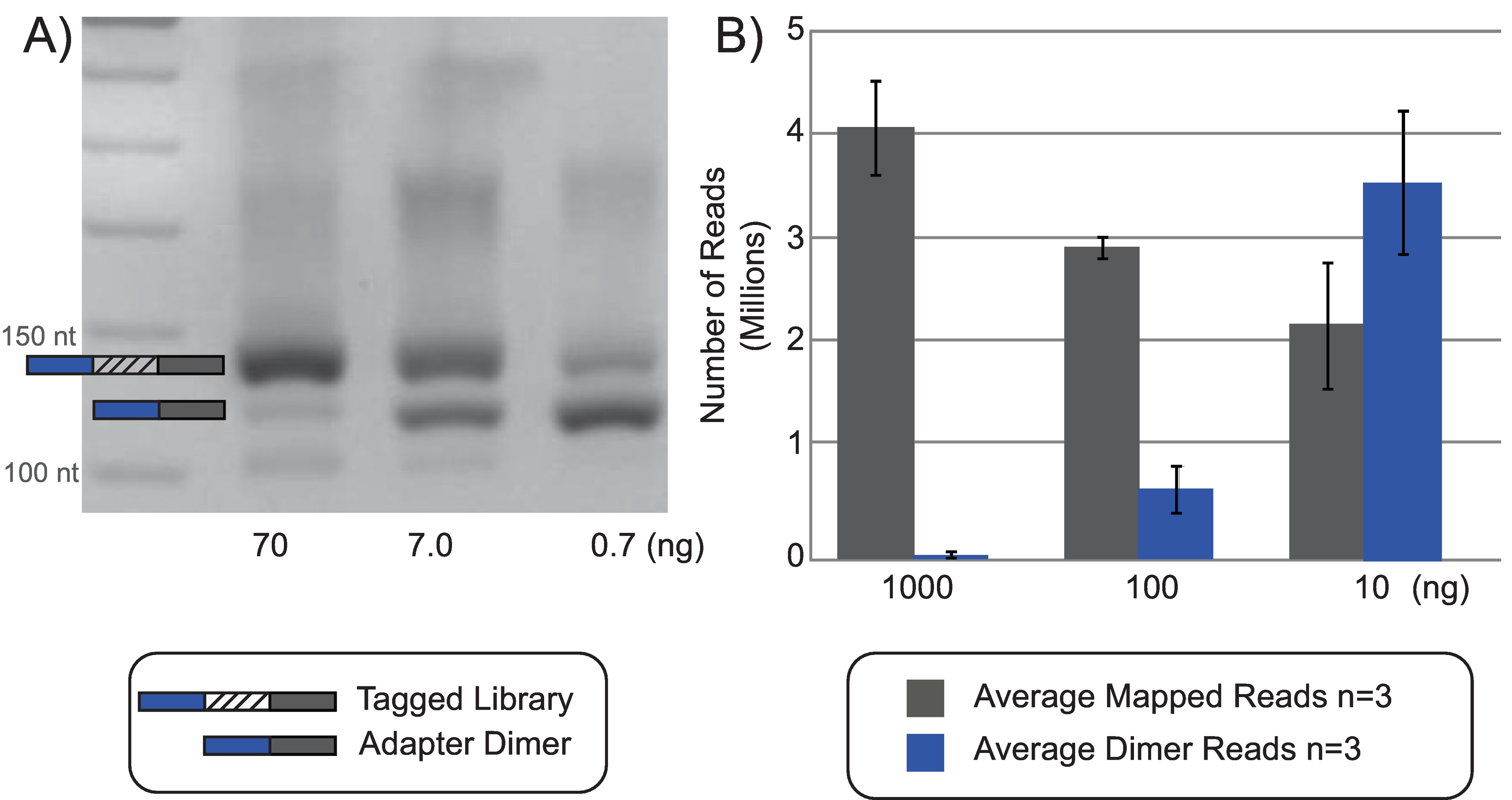

Supplement: S1 Fig — A) 4% agarose gel analysis of bead purified PCR products. Libraries were prepared with unmodified adapters and 0.7 to 70 ng of a synthetic miRNA (Let 7d-3p (NNN)). B) NGS data showing average number of mapped reads and average number of adapter dimer reads. Libraries were prepared with the recommended conditions of the TruSeq Small RNA Library Prep Kit using unmodified adapters and brain total RNA at inputs of 10, 100, and 1000 ng. Data analysis was performed using Geneious. (TIF) [file pone.0167009.s001.tif]

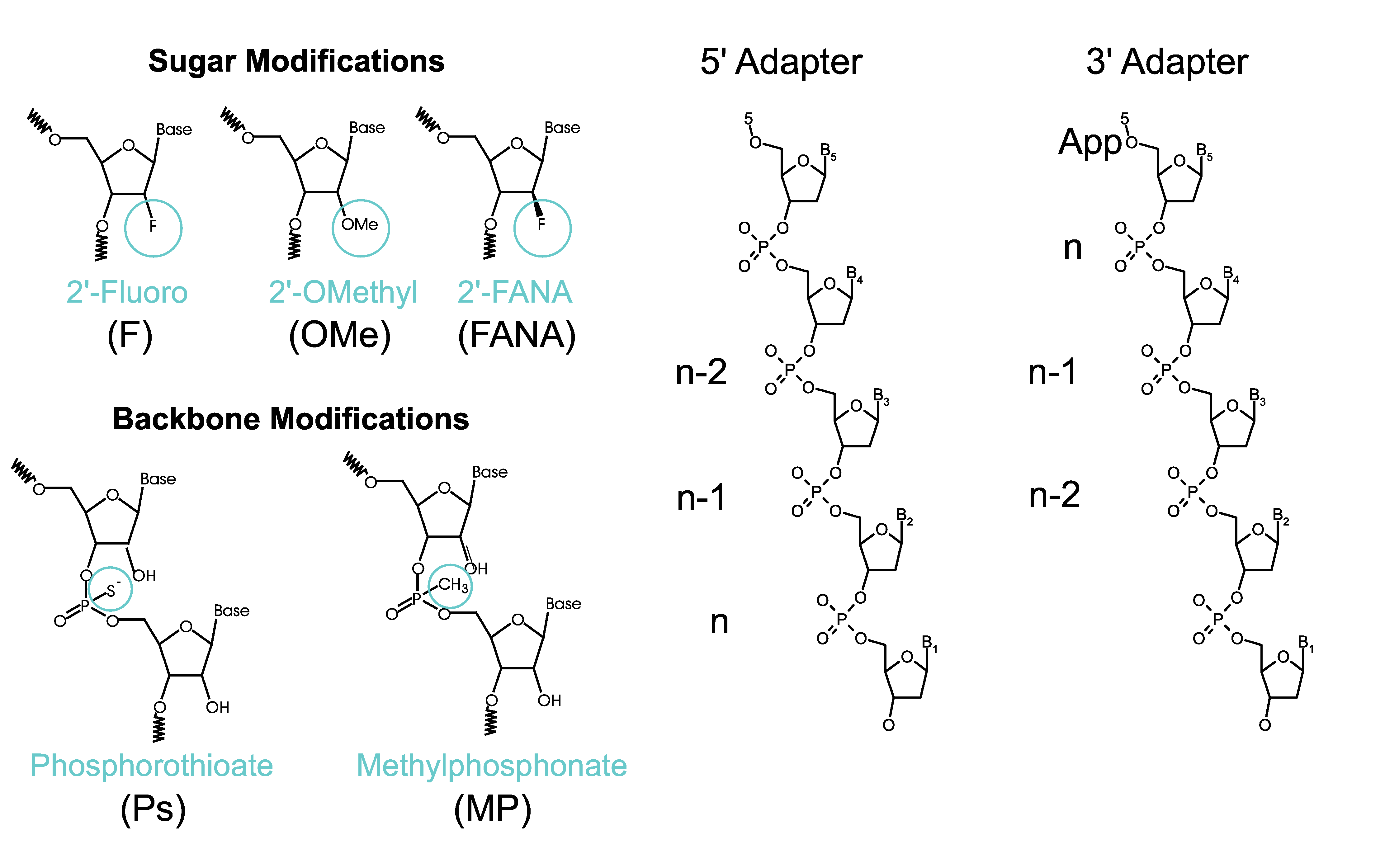

Supplement: S2 Fig — Representative chemical modifications screened on adapter oligonucleotides for sRNA-Seq library preparation. Nomenclature for the positions modified is shown. (TIF) [file pone.0167009.s002.tif]

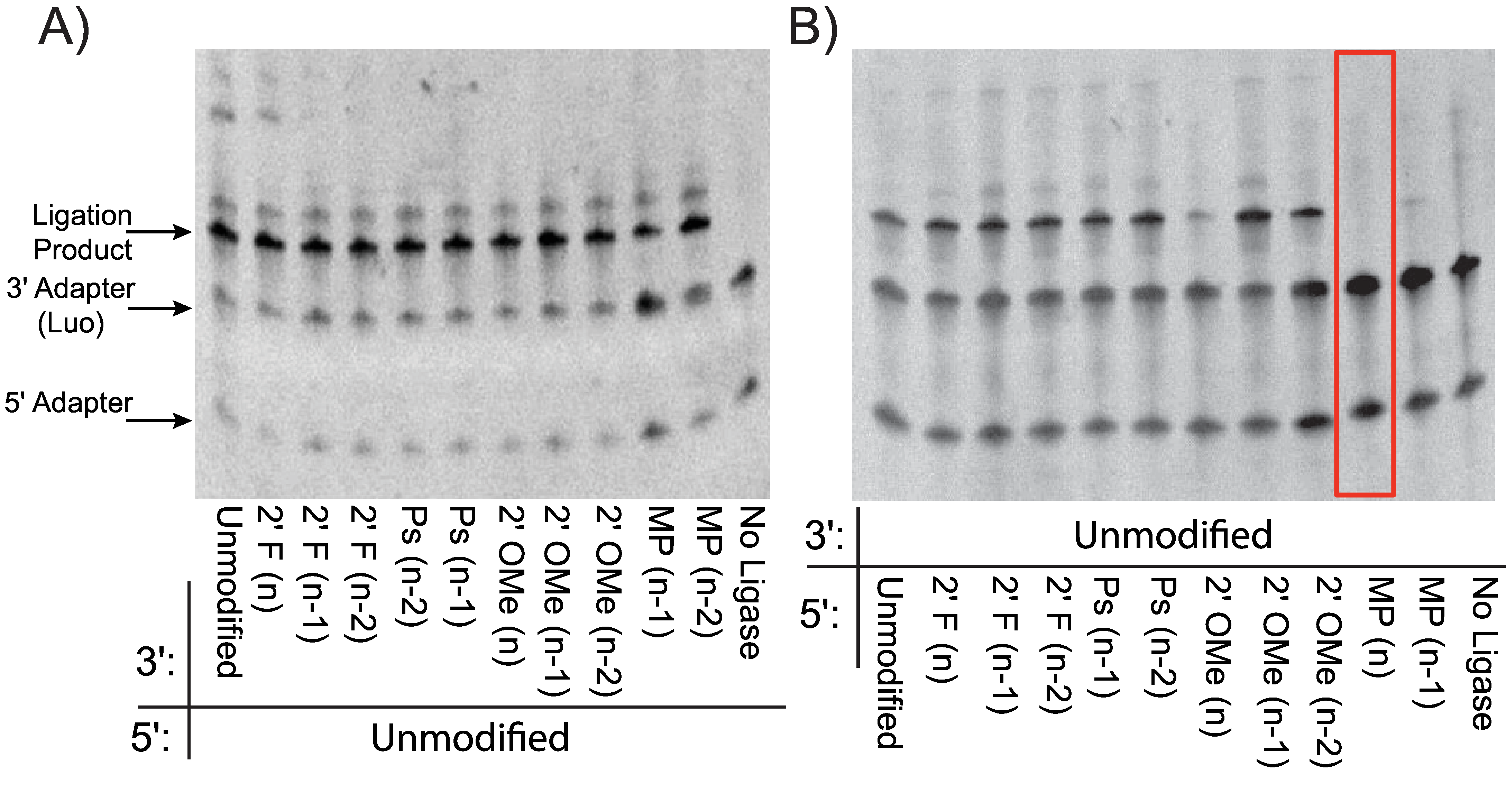

Supplement: S3 Fig — Example of screened modifications on A) 3´ adapter Luo or B) the 5´ adapter for ligation efficiency. Red box indicates a modification in which ligation to the substrate was undetectable. Reactions were incubated with 10 U T4 RNA Ligase 1 for 1 hour at 37°C. (TIF) [file pone.0167009.s003.tif]

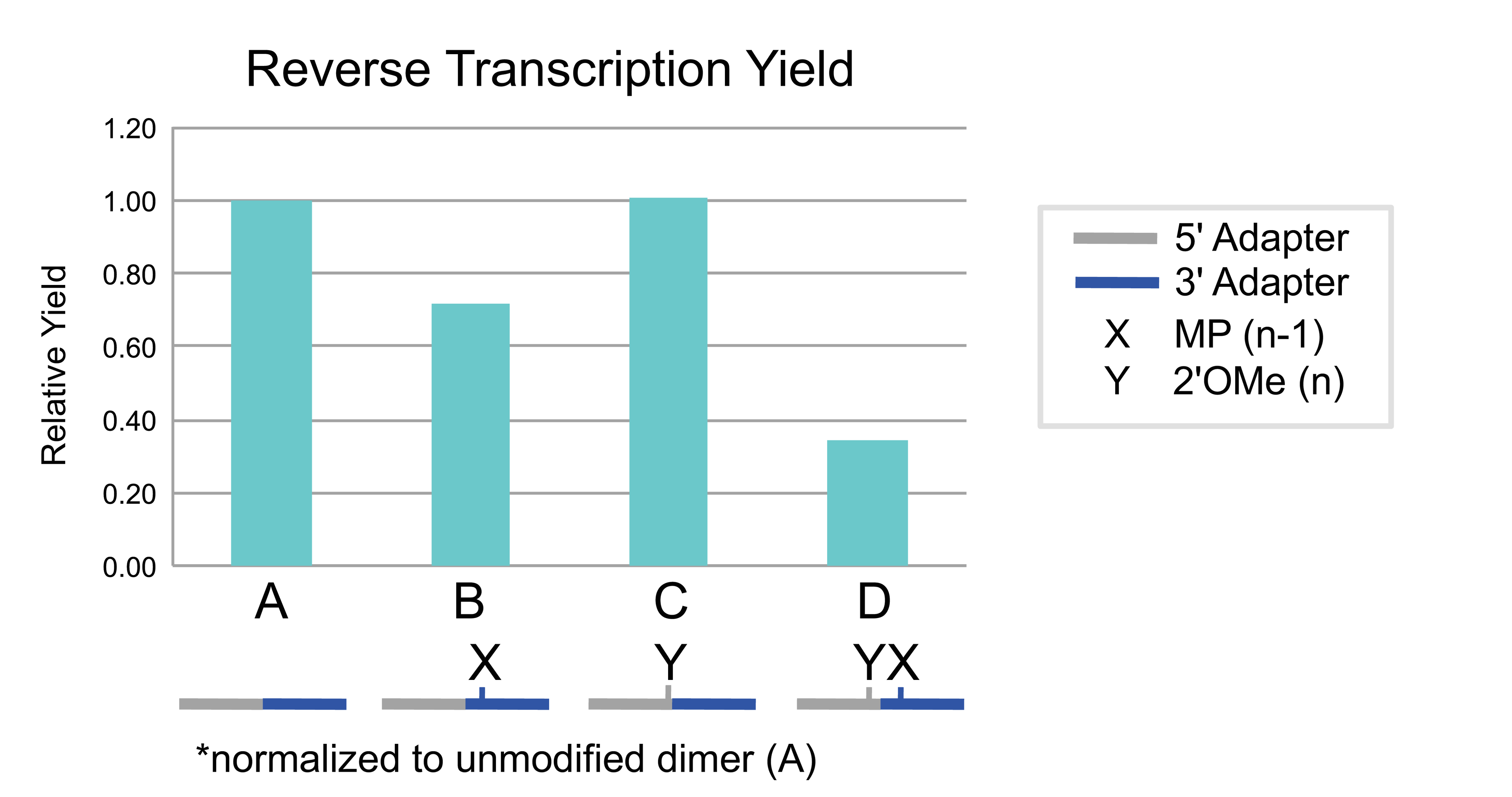

Supplement: S4 Fig — Reverse transcription was performed on different modified adapter dimer substrates using a FAM-labeled RT primer. Ligation product from unmodified adapters served as the control for normalization (column A). Read through from a single modification on either the 3´ adapter (B) or 5´ adapter (C) was compared to a double modified substrate (D), both 5´ and 3´ adapters modified. RT products were run on a gel, imaged, and quantified for relative cDNA synthesis yield determination. (TIF) [file pone.0167009.s004.tif]

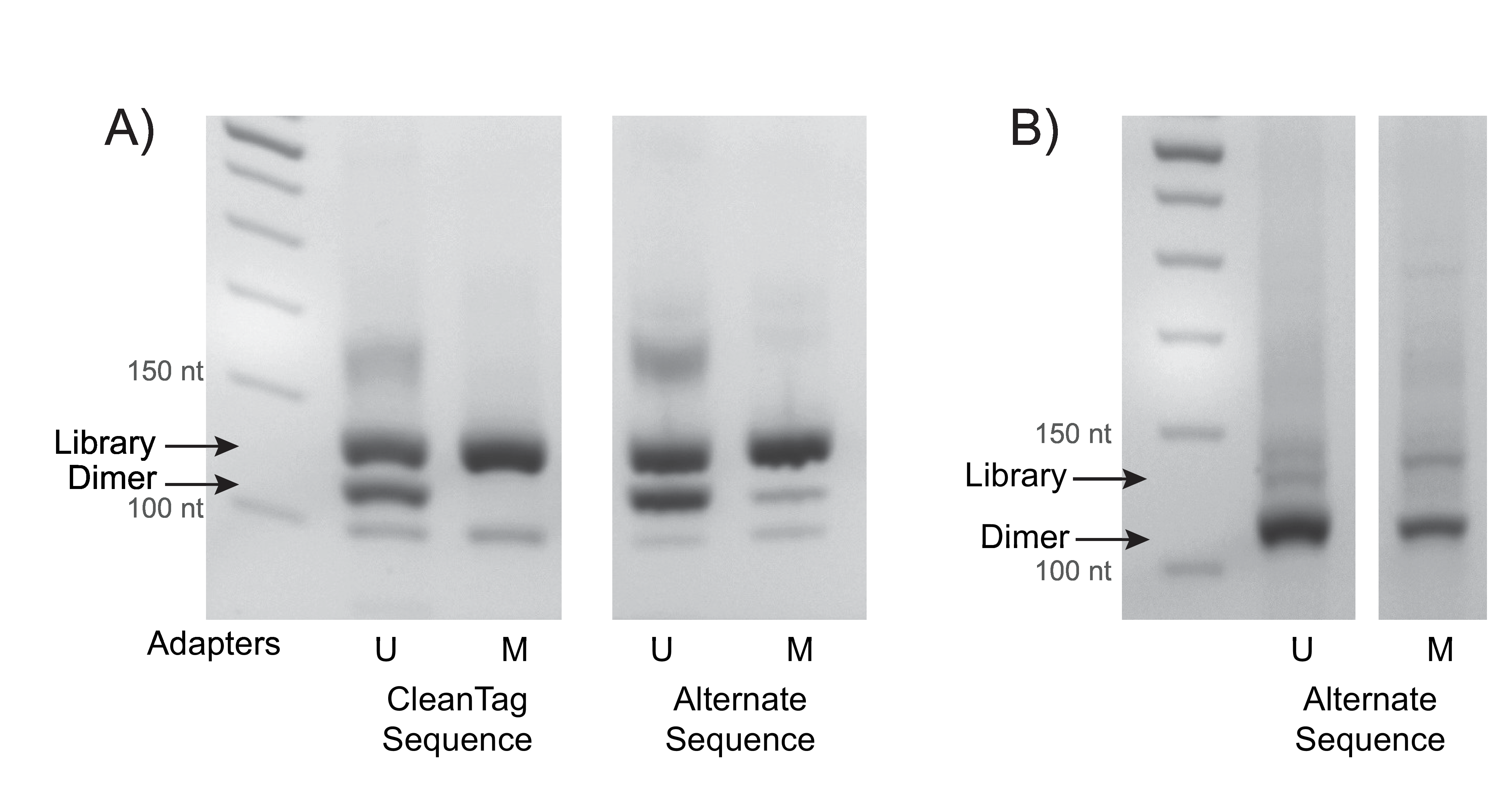

Supplement: S5 Fig — A) Library preparation using 7 ng synthetic miRNA (Let 7d-3p (NNN)) input. U = both adapters were unmodified; M = both adapter were modified with top modifications. The CleanTag adapter set was compared to an alternate adapter set with a different sequence for the 3´ adapter. The same modifications were used for the alternate set. B) The alternate adapters were also tested with 1000 ng brain total RNA input. The CleanTag library preparation workflow was used to prepare libraries. (TIF) [file pone.0167009.s005.tif]

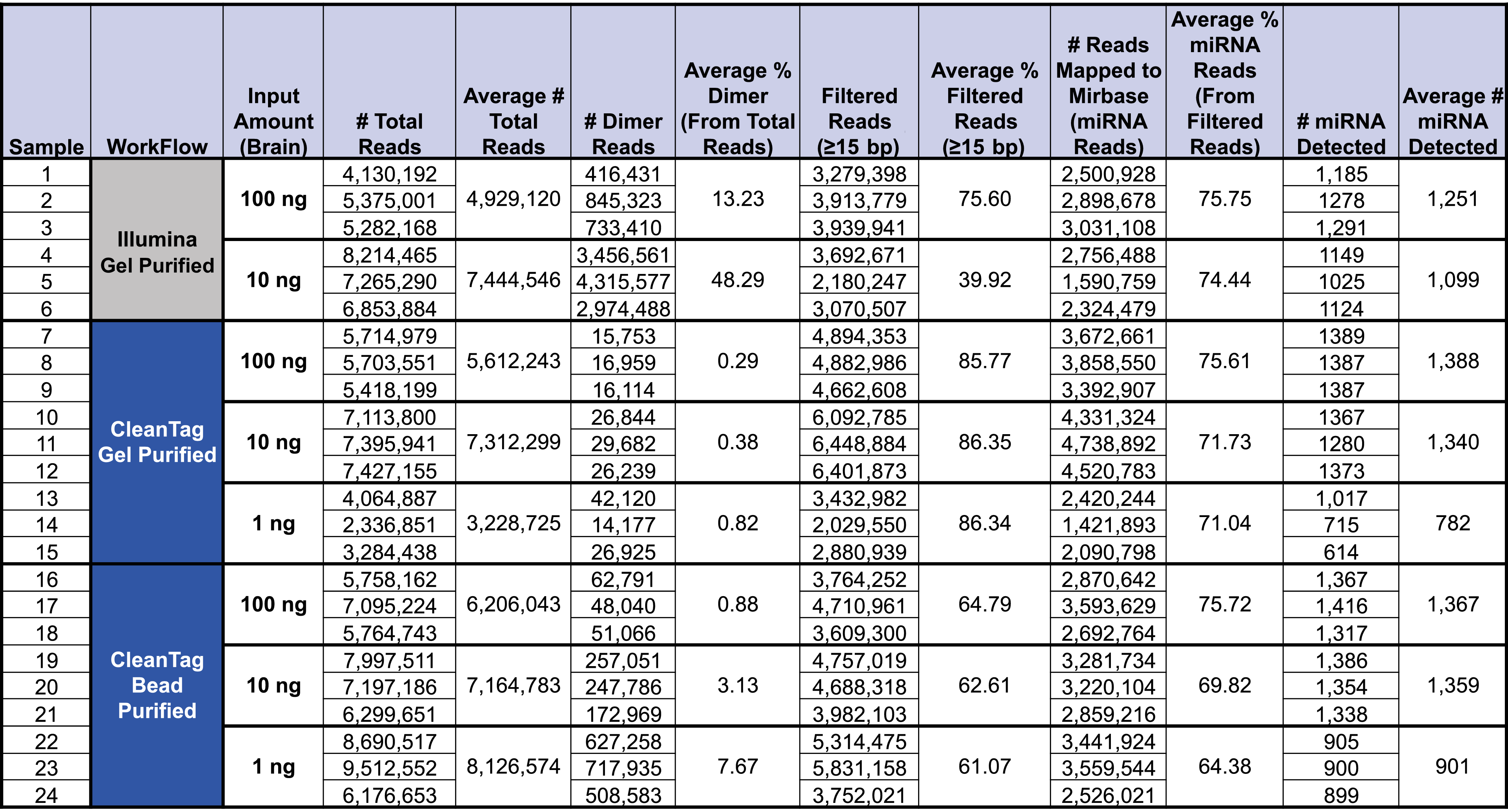

Supplement: S2 Table — Comparison of data between different small RNA library preparations using human brain total RNA input between 1–100 ng. Libraries sequenced on HiSeq 2500 SR, 1x 100bp. Data analysis was performed using Geneious. Samples run in triplicate. (TIF) [file pone.0167009.s008.tif]

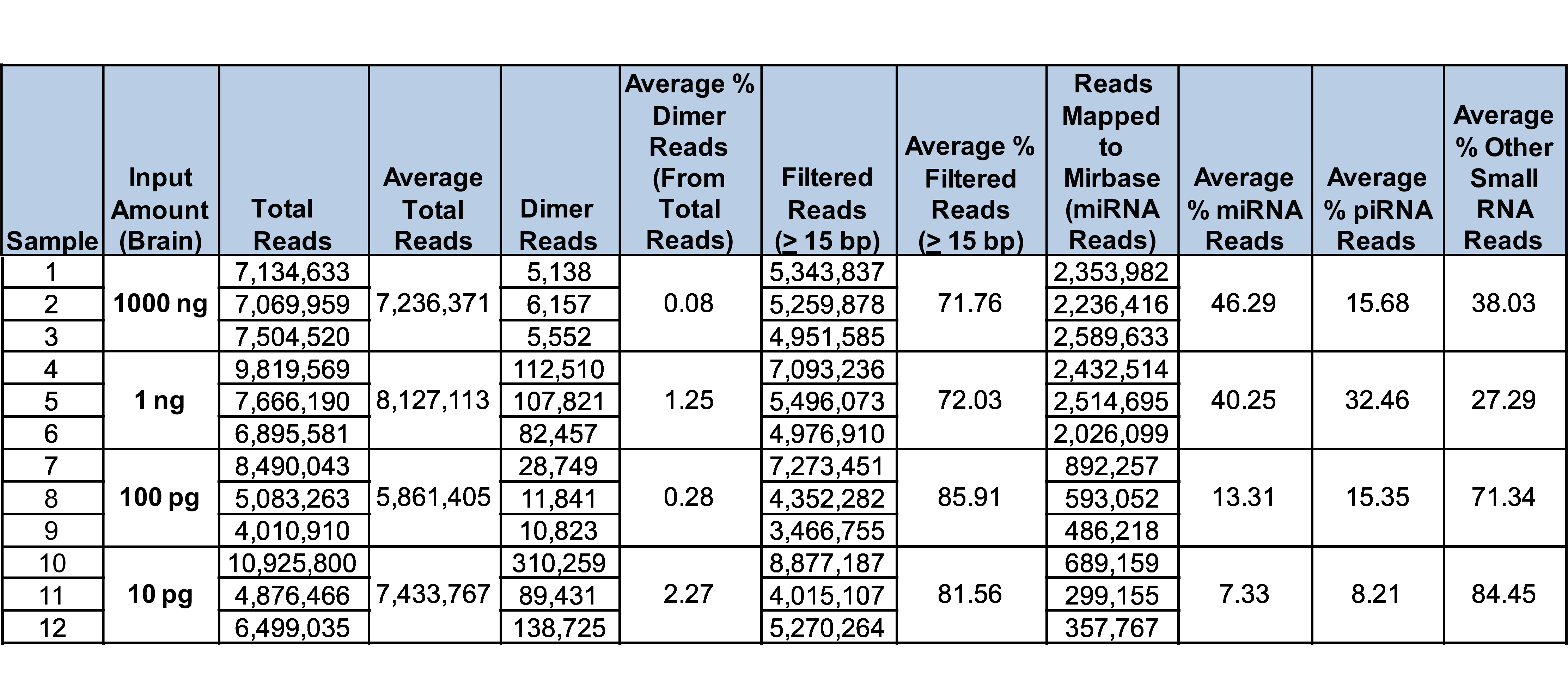

Supplement: S3 Table — Libraries prepared with human brain total RNA at various input amounts using CleanTag library preparation kit. All samples performed in triplicate. Inputs at 1000 and 1 ng were bead purified. Inputs at 100 and 10 pg were pooled and gel purified. Libraries sequenced on HiSeq 2500 SR, 1x 100bp and data analysis was performed with Galaxy. (TIF) [file pone.0167009.s009.tif]

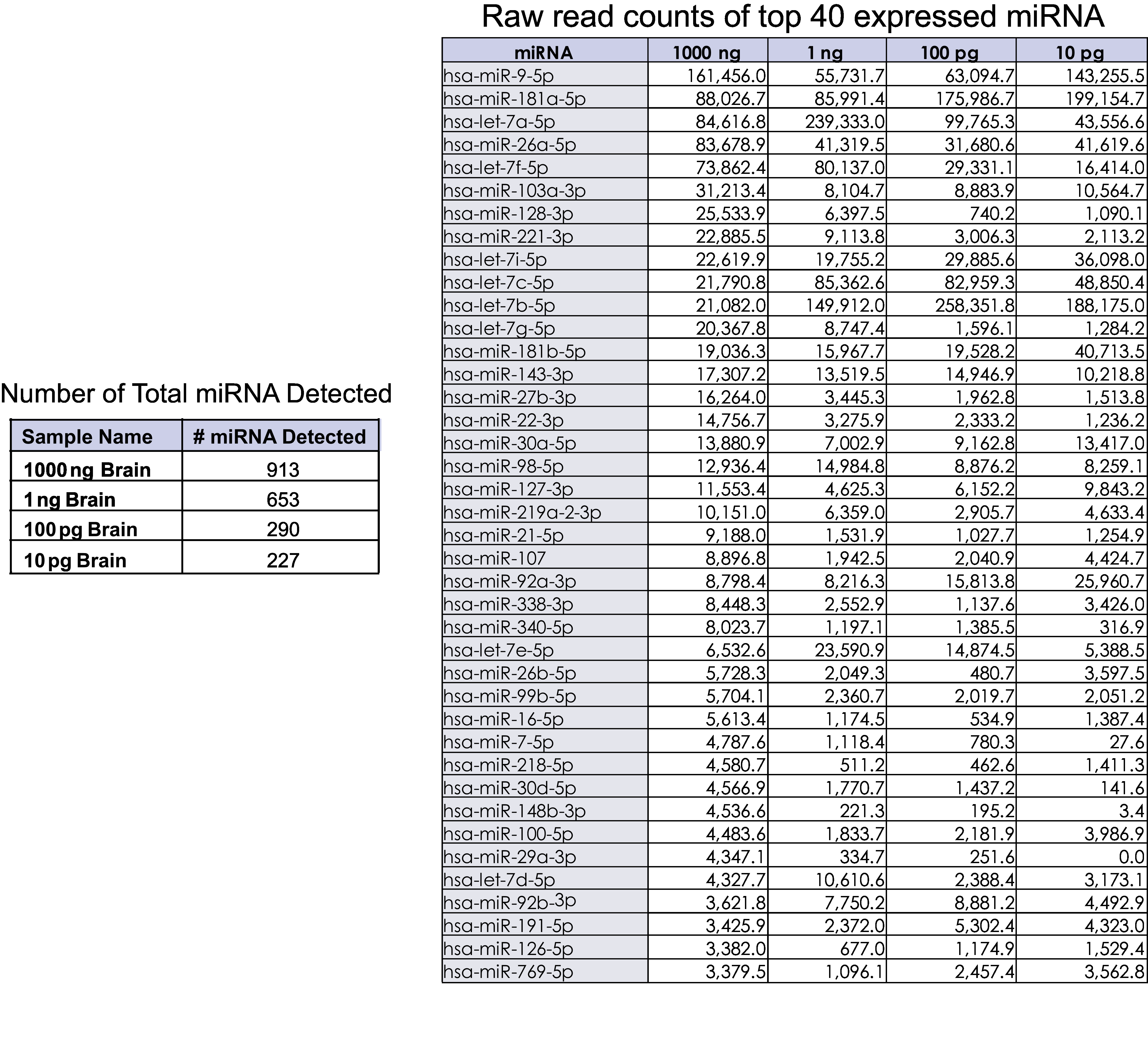

Supplement: S4 Table — Breakdown of data from S3 Table shows the average total number of miRNA detected at each input and the average raw read counts for the top 40 expressed miRNA at each input. Data analysis was performed with Galaxy. (TIF) [file pone.0167009.s010.tif]
