# Supplementary material for: Small RNA Library Preparation Method for Next-Generation Sequencing Using Chemical Modifications to Prevent Adapter Dimer Formation
Source: PLoS One. 2016 Nov 22;11(11):e0167009. doi: 10.1371/journal.pone.0167009 (PMC5119831; doi:10.1371/journal.pone.0167009)
Supplement: S1 File — (PDF) [file pone.0167009.s006.pdf]

S1 File

Analysis of Bioanalyzer Peaks Generated From sRNA Library Preparation

We explored the origin of any major peaks shown in a bioanalyzer trace to ensure lower molecular weight peaks would not amplify on the flow cell and to identify the variety of small RNA species tagged in this workflow. Further investigation revealed specific identities for many of the peaks in the bioanalyzer trace **(S1 File Fig1)**. The following peak sizes correspond to single stranded oligonucleotides as demonstrated by running individual oligonucleotides in water on a BioAnalyzer high sensitivity DNA chip: RT Primer (33 nt), 35 nt Marker (35 nt), 3′ Adapter (40 nt), 5′ Adapter (42 nt), Forward PCR Primer (73 nt), and Reverse PCR Primer (82 nt) **(S1 File Fig2)**.

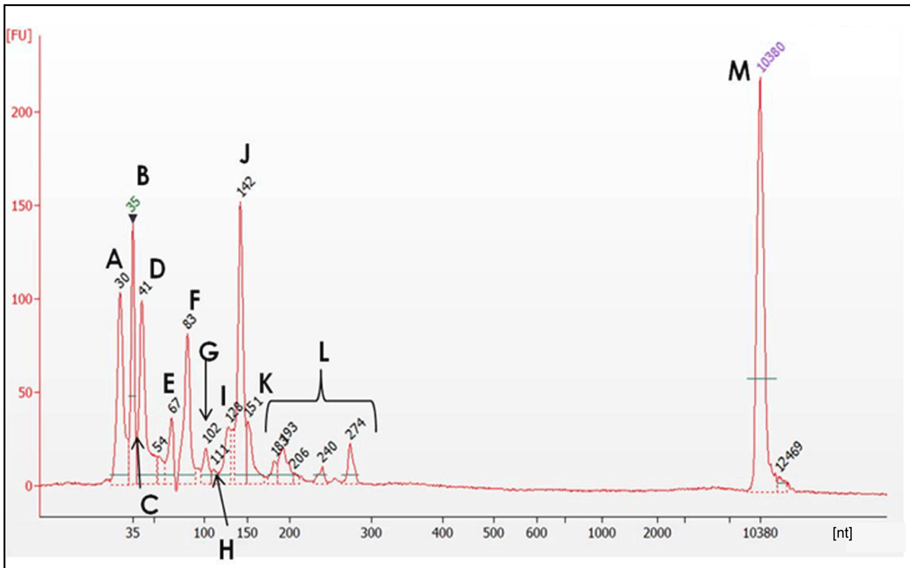

A RT primer (30)
B 35 bp marker (35)
C 3' Adapter (39)
D 5' Adapter (41)
E Forward primer (67)
F Reverse primer (83)
G Unproductive library (miRNA) (102)
H Unproductive library (other sRNA) (111)
I Degraded RNA (130)
J miRNA (140)
K piRNA/other small RNA (150)
L Undetermined- other small RNA, genome, transcriptome, lncRNA (180-274)
M Upper Marker (10,380)

S1 File Fig1. An overview of peaks in a bioanalzyer trace for small RNA library prep.

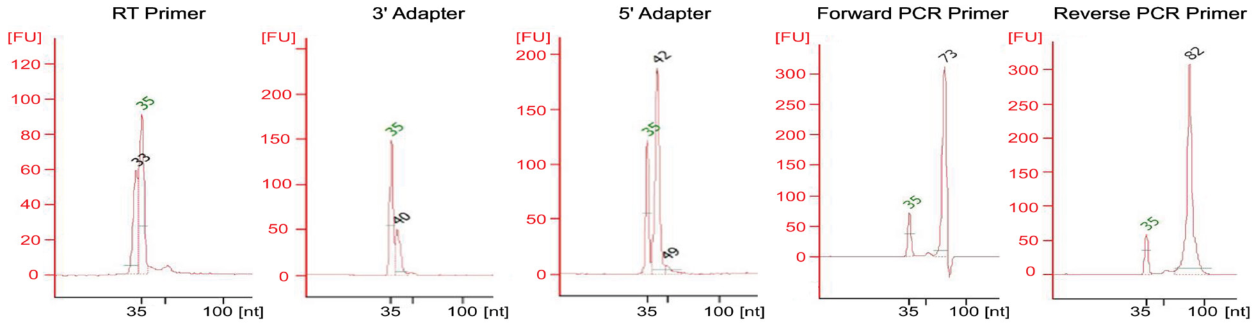

S1 File Fig2. BioAnalzyer trace of the migration of single stranded primer and adapter peaks. Oligonucleotides in water loaded onto high sensitivity DNA BioAnalyzer chip.

The RT primer generally runs prior to the 35 nt bioanalyzer marker which can sometimes shift the bioanalyzer traces so it is important to manually set the lower marker as the second peak if there are 3 initial peaks. The 3′ Adapter often gets masked by the 35 nt marker and other nearby peaks, especially since its concentration is so low. The single stranded oligonucleotides also do not run true to size but instead run consistently higher by 10-20 nt. For example, the RT primer is a 22 nt oligonucleotide but runs at 31 nt while the forward PCR primer is a 50 nt oligonucleotide that runs at 73 nt on the bioanalzyer. The next peak in question runs at 100 nt and was determined to be adapter tagged miRNA libraries that have been PCR amplified with a forward primer and excess RT primer **(S1 File Fig3)**.

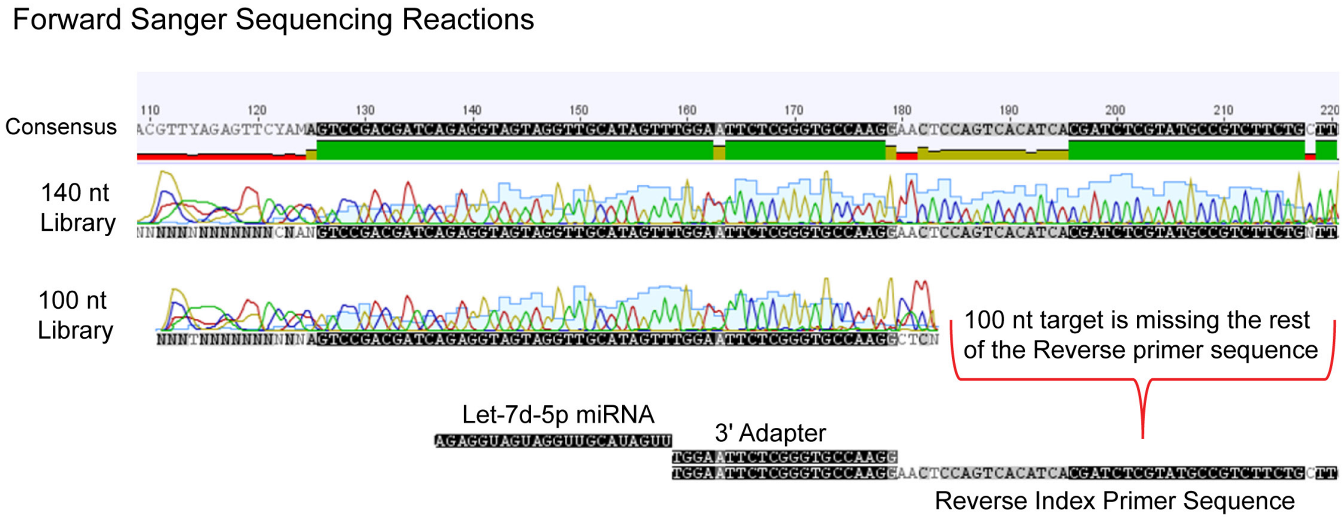

S1 File Fig3. Sanger sequencing traces of full length miRNA library (140 nt) and a truncated miRNA library (100 nt). Library preparation was done using a synthetic Let7d miRNA and bands on a gel were cut out that correspond to full length miRNA library (140 nt) and predicted truncated miRNA library (100 nt). These samples were purified and submitted for Sanger Dideoxy sequencing.

To prove this, we prepared libraries with a synthetic 22 nt oligonucleotide, ran an agarose gel, and cut out bands at 100 (side product) and 140 nt (library). These samples were subjected to Sanger dideoxy sequencing and results revealed that the 100 nt side product was lacking the extra 40 nt contributed from the reverse PCR primer. Because this side product lacks a region of the reverse PCR primer it will not be able to bind the flow cell primers on that side and is also without a barcode. Increased levels of RT primer drive this reaction so it is important to optimize RT primer levels to

prevent loss of miRNA to unproductive libraries **(S1 File Fig4)**. This truncated library side reaction can vary considerably from one reaction to the next. The undesired adapter dimer side product runs at 120 nt and is a peak that is often not present in the CleanTag workflow. Next there is a peak which runs as a small shoulder around 130 nt directly in front of the main miRNA library peak. We speculate that this peak is due to degraded RNA species that are around 10 nt in length. These fragments typically get filtered out in bioinformatic analysis because they do not meet the length threshold of 15 nt and are considered “junk”. It is unclear whether these could actually be smaller distinct RNA species of regulatory importance [2]. Tagged miRNA libraries that have been amplified with forward and reverse PCR primers run at 140 nt while the 150 nt peak contains several tagged small RNA types such as piRNA, tRNA etc. The 150 nt peak can vary in amount significantly from one sample type to the next and from one library preparation to the next even within the same sample type. The region of 180-275 nt is less characterized and we tentatively attribute these peaks to be potential longer fragments of the transcriptome. Overall, it is important when analyzing bioanalayzer traces to observe a pattern rather than specific peak sizes. Sizes of peaks can shift on a bioanalzyer trace by 10-20 nt in any given chip run.

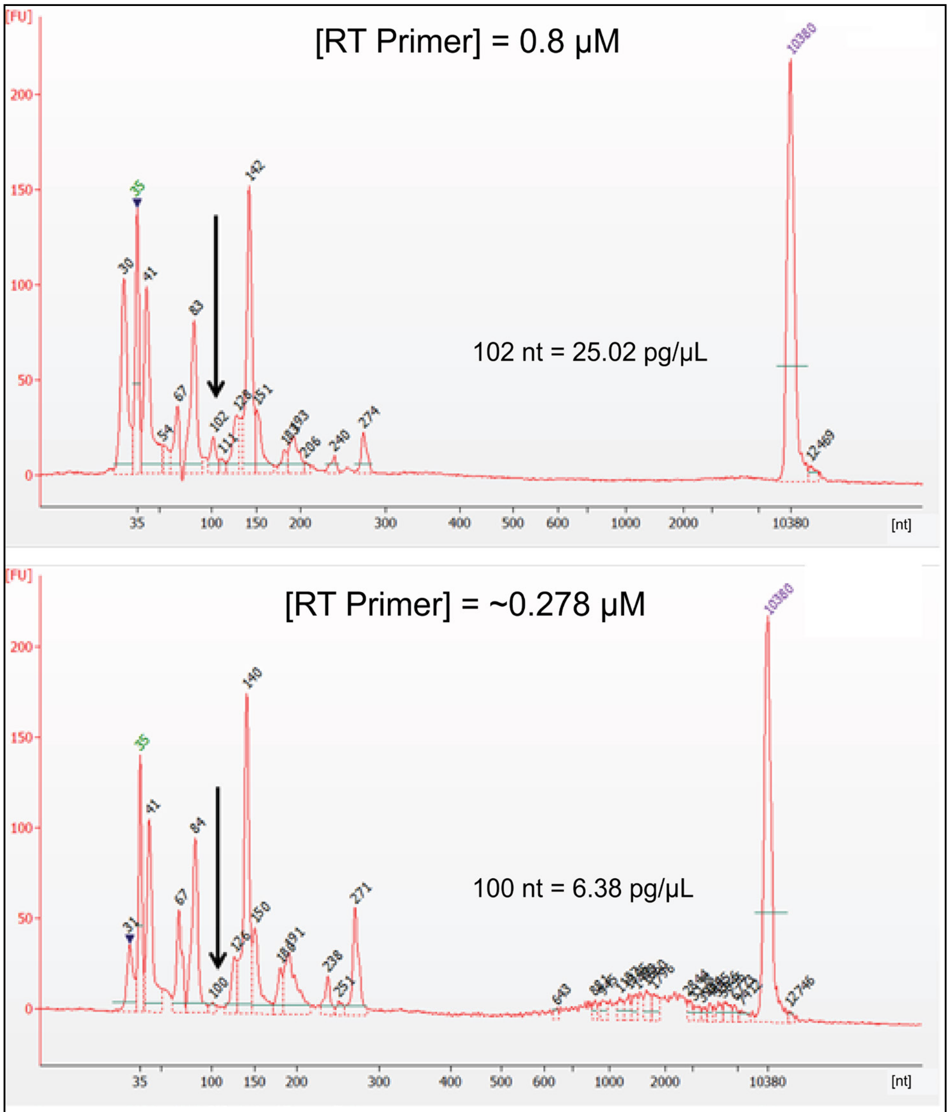

S1 File Fig4. High RT primer concentration causes non-productive library peak. Bioanalyzer traces of crude small RNA libraries prepared using 1000 ng human total brain RNA input with CleanTag workflow. A) High RT primer concentration results in more truncated library formation at 100 nt. B) Lower RT primer concentration results in a decrease of truncated library formation.
